# Combining Time‐Stamped Insect Sampling With eDNA‐Metabarcoding of Guano to Reconstruct Community Interactions

**DOI:** 10.1002/ece3.73509

**Published:** 2026-04-17

**Authors:** Tamara R. Hartke, Manus Wittenhorst, Martin Koch, Christoph Scherber, Sönke Twietmeyer, Sarah J. Bourlat, Michel Böhme, Ameli Kirse

**Affiliations:** ^1^ Leibniz Institute for the Analysis of Biodiversity Change (LIB), Center for Biodiversity Monitoring and Conservation Science Bonn Germany; ^2^ BioConsult SH GmbH & Co. KG Husum Germany; ^3^ Department of Biogeography Trier University Trier Germany; ^4^ Bonn Institute for Organismic Biology University of Bonn Bonn Germany; ^5^ Department of Research and Documentation Eifel National Park Schleiden Germany

**Keywords:** biodiversity monitoring, bulk samples, chronoecology, eDNA metabarcoding, *Plecotus*, species interaction monitoring

## Abstract

Understanding the temporal dynamics of predator–prey interactions is the basis for predicting behavioral responses to climate or other environmental changes. Bats, as generalist apex predators, can provide a holistic view on ecosystem health, because their diet integrates signals from diverse insect communities and rapidly reflects environmental change. However, most molecular diet studies lack the temporal resolution needed to infer hunting strategies and prey choice mechanisms, because guano‐derived species lists cannot be meaningfully interpreted without knowing when and what potential prey were available. Previous research has typically analyzed eDNA metarcoding data or insect activity patterns separately, limiting insights into mechanisms underlying prey selection. This study combines eDNA metabarcoding of bat guano with metabarcoding of time‐stamped insect samples collected using automated Malaise traps to investigate the foraging ecology of long‐eared bats (*Plecotus* sp.). Guano‐derived prey communities most closely resemble insect bulk samples captured between 22.00 and 6.00, with species‐specific differences in peak foraging times and prey composition. We further detected distinct prey‐selection patterns between two closely related species, *Plecotus auritus* and *Plecotus austriacus* , consistent with their individual hunting strategy. Our findings demonstrate that adding a temporal axis to molecular diet analysis enables direct inference of prey selection and foraging behavior from guano. This integrative approach advances biodiversity monitoring and offers a tool box for conservation planning under changing environmental conditions. Our findings demonstrate that adding a temporal axis to molecular diet analysis enables direct inference of prey selection and foraging behavior from guano. This integrative approach advances biodiversity monitoring and offers a tool box for conservation planning under changing environmental conditions.

## Graphical abstract

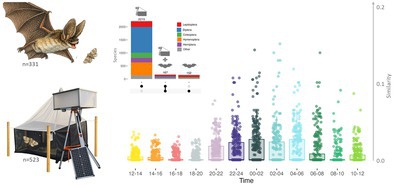

## 1 Introduction

As anthropogenic pressures change community composition at multiple trophic levels, understanding interspecific relationships will be vital to the success of conservation and restoration efforts. Monitoring species interactions, such as predator–prey or plant‐pollinator interactions, to elucidate the impacts of human activities on overall ecosystem health and stability will require multispecies and hence multimodal or multilevel approaches which can deliver fine‐grained information over time and space (Kirse et al. 2025). Modern technologies and approaches have emerged to meet the growing demand for rapid, standardized species identification that this requires. One particularly flexible tool is DNA metabarcoding and its extension environmental DNA (eDNA) metabarcoding, which uses DNA extracted from environmental sources such as soil, water, feces, or air (Ruppert et al. 2019; Taberlet et al. 2012).

Metabarcoding facilitates the simultaneous identification of thousands of samples, significantly accelerating the process of species identification, providing reference libraries are complete (Buchner et al. 2025; Chua et al. 2023). Although DNA metabarcoding can generate species presence‐absence lists that provide valuable insights into the status of the associated ecosystem, it is not yet possible to obtain reliable abundance data through this method and ecologically important information remains missing (Sickel et al. 2023). Importantly, eDNA metabarcoding is noninvasive and can, depending on source material, provide insights into species interactions, such as food webs and predator–prey dynamics (D'Alessandro and Mariani 2021; Deagle et al. 2023). For instance, predator feces can reveal dietary preferences, shed light on local prey communities (Ashrafi et al. 2013), and offer information about a predator's ecological role, behavior, and contributions to ecosystem services (Ancillotto et al. 2022; Sickel et al. 2023).

As insect apex predators, bats can be particularly valuable for eDNA‐based biodiversity and ecosystem monitoring, and eDNA metabarcoding of bat guano has already yielded new insights into bat prey spectra (Hurpy et al. 2025; Scholz et al. 2025). However, many aspects of bat foraging behavior, particularly temporal patterns, remain poorly understood. This includes the effects of relatively simple factors such as insect abundance and behavior (Dietzer et al. 2024; Vekhnik et al. 2025) or land use (Scholz et al. 2025).

Among insectivorous bats, the gray long‐eared bat ( *Plecotus austriacus* ) and the brown long‐eared bat ( *Plecotus auritus* ) are notable for their distinct foraging strategies and adaptability (Dietz et al. 2007; Entwistle et al. 1996). These sympatric species predominantly feed on moths and other flying insects (Andriollo et al. 2021), often in highly structured environments. *Plecotus* are known to not only hunt in flight but also to glean prey from foliage or bark. Like many other bat species, they feed primarily on lepidopterans, but due to their hunting strategy, can also have a relatively high proportion of nonflying insects in their diet (Ashrafi et al. 2011). Previous molecular studies show that lepidopterans account for 40%–90% of species in the *Plecotus* diet (Andriollo et al. 2021; Ashrafi et al. 2011; Razgour et al. 2011), with *P. auritus* at the lower end of that range (40%–60% Lepidoptera) and *P. austriacus* more specialized (85%–90%). Earlier reports of 27.8% nonflying prey for *P. auritus* and 2.7% for *P. austriacus* indicate a greater reliance on gleaning by *P. auritus* , while *P. austriacus* is a more typical aerial feeder (Andriollo et al. 2021; Ashrafi et al. 2011), suggesting niche differentiation.

While some inferences can be drawn from guano metabarcoding alone, understanding foraging decisions and prey preferences requires simultaneous assessment of the potential prey community. A variety of insect trapping methods have been developed, with different strengths and weaknesses. One of the most frequent types is the Malaise trap, which effectively captures a wide range of flying insects (Geiger et al. 2016) but requires intensive maintenance. Temporal resolution of Malaise traps is often limited, as it requires manual bottle changes. Consequently, samples are typically collected over extended periods, often spanning several weeks or months, which precludes detailed analysis on direct interactions or circadian species activity patterns. To address this limitation, automated time‐controlled Malaise traps, such as the AMMOD Multisampler, have been developed (Kirse et al. 2024). The Multisampler rotates between 12 collection bottles at predefined time intervals, enabling fine‐scale temporal resolution of insect activity. Traditionally, identification of such multi‐taxon insect samples has required multiple group‐specific taxonomic experts, making the process highly time‐consuming and costly, especially in face of the growing shortage of skilled taxonomists (Buchner et al. 2025). Metabarcoding insect bulk samples produces relatively rapid, inexpensive species lists which can be directly compared to those generated from metabarcoding of guano samples. In this way, knowledge of species interactions can be increased by integrating multiple technologies (Kirse et al. 2025), approaches and, in the case of metabarcoding, various source substrates (Thomas et al. 2024).

Here we go beyond distinguishing the different hunting strategies of two closely related *Plecotus* bat species through metabarcoding of their guano to investigate their interactions with local insect populations. We integrate molecular‐ and technology‐based methods to examine predator–prey relationships of long‐eared bats (*Plecotus* spp.), linking prey community composition and activity patterns to dietary preferences. We analyzed eDNA from guano collected from four Plecotus bat colonies over a 3‐month period in the Eifel region of western Germany. We compared these data with insect samples collected with automated Malaise traps installed in the colonies' hunting grounds, which were verified through acoustic monitoring. We hypothesize that:

1. eDNA metabarcoding of bat guano detects similar insect community compositions as those captured by an automated Malaise trap during the night.
2. eDNA metabarcoding of bat guano, in combination with time‐controlled Malaise traps, enables conclusions about the preferred hunting times of different bat species.

This research advances species interaction monitoring by combining state‐of‐the‐art molecular and automated insect traps to provide a more comprehensive understanding of bat foraging ecology. By linking detailed prey availability data with temporal foraging patterns, we aim to contribute to the conservation of these bat species and the ecosystems they inhabit.

## 2 Material and Methods

### 2.1 Sampling Area

**FIGURE 1:**
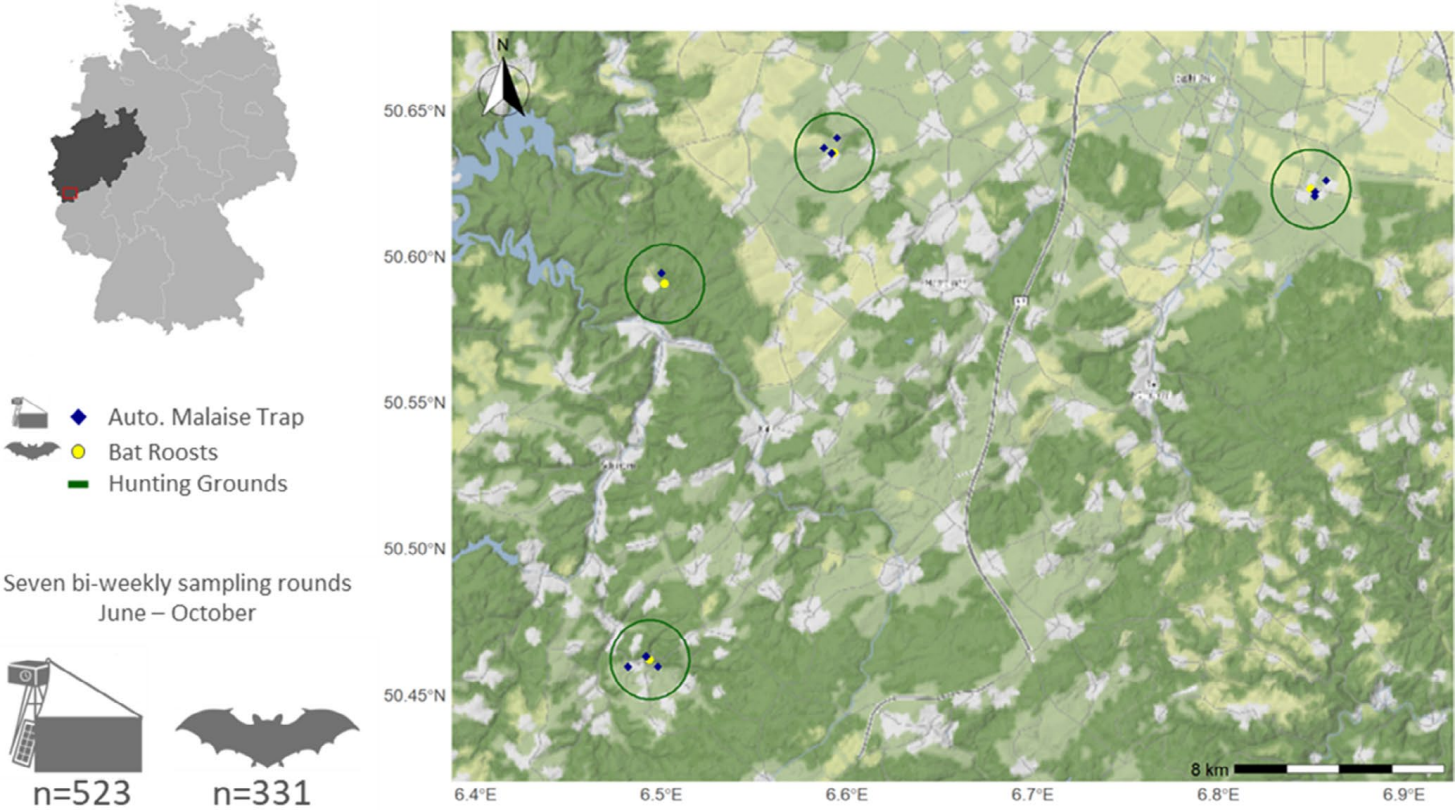
Geographic location of the four *Plectous* sp. colonies in the Eifel region, North‐Rhine‐Westphalia, Germany. Roosts, indicated as yellow circles, are located in the centers of the solid green circle, indicating an expected flight radius of 1500 m. Insect trap locations are indicated as blue diamonds. Over the seven sampling rounds, 523 time‐stamped insect bulk samples and 331 guano samples were collected.

The study was conducted in the Eifel low mountain range in southwestern North‐Rhine‐Westphalia (NRW), Germany (Figure 1). The four sampling areas were situated in the Rur‐Eifel and Zülpicher Börde regions of NRW, east of Cologne and Bonn near the Belgian border (Table S1).

Each area hosted a colony of bats of the genus *Plecotus*, including two *P. auritus* and two *P. austriacus* roosts. One *P. auritus* roost was located in an old forester's lodge near the village Schleiden‐Wolfgarten. The area is known as Kermeter and has been part of the Eifel National Park since 2004 (henceforth Eifel National Park, NP). A second colony of *P. auritus* inhabited the nave of the church in Wildenburg Castle (WI). One *P. austriacus* roost was located within the roof truss of St. Peter's Church in Mechernich‐Berg (Berg, BE) and the second was situated within the nave of the evangelical church in Flamersheim (FL). Between June and October 2022, bat guano collection and Malaise trap catches were coordinated into 7 sampling rounds at 14 day intervals, such that guano and insect bulk catches were collected on the same day. Due to delays in accessing the roosting location, sampling in Wildenburg began 2 weeks later than the other areas.

#### 2.1.1 Verification of Bat Roosts & Hunting Grounds

Infrared camera traps pointed toward the ceiling of each roost verified presence of *Plecotus* (Figure S1). During sampling round six (31 August 2022 to 13 September 2022), AudioMoth recorders (Hill et al. 2019) were attached to each Multisampler to confirm *Plecotus* at the potential hunting grounds. Audio recordings were organized and pre‐analyzed with Batscope4 (Obrist and Boesch 2018). All bat calls not classified as *Pipitrellus* were manually inspected with Avisoft‐SASLab‐Lite audio analysis tool. Because the calls of *Plecotus* and *Pipitrellus* differ substantially, removing *Pipitrellus* calls from the acoustic dataset is highly unlikely to result in false negatives for *Plecotus*. Subsequently, the remaining calls were identified to species or species group following Russ (2012) and Barataud (2015). Additionally, mist‐netting was conducted at one hunting ground in the Eifel Nationalpark as part of long‐term bat monitoring and found *P. auritus* hunting regularly near the malaise traps. Two mist‐netted adult females were fitted with a 0.3 g vhf‐transmitter (Holohil), leading us to their maternity roost.

#### 2.1.2 Guano Sampling

To collect guano, sheets of paper were laid underneath the roosts. Every two weeks, 20–40 guano pellets per site were individually transferred into 1.5 mL Eppendorf tubes using flame‐sterilized and DNA AWAY (Thermo Fisher Scientific, Waltham, MA, USA) washed tweezers. After sampling, paper sheets were replaced to avoid contamination between sampling rounds. Collected pellets were stored at −20°C until further processing.

#### 2.1.3 Malaise Trapping

At the beginning of June 2022, we set up eleven Malaise traps equipped with an automated Multisampler (Kirse et al. 2024; Figure S2). A rotary mechanism housed inside the Multisampler holds 12 bottles and changes them at predetermined intervals. Here, sample bottles rotated every 2 h, resulting in a 24‐h cycle preserving circadian patterns of insect activity (Kirse et al. 2025).

Malaise traps were set up in potential bat hunting grounds like orchards, meadows, and marshes within 1500 m of the roost (Figure 1). In FL, BE, and WI, traps were set up at three different locations. In the Eifel National Park, two Malaise traps were installed. Trapping liquid was 96% denatured ethanol (1% MEK, type 641) to ensure preservation of DNA. Collection bottles were replaced in a 14‐day cycle corresponding to guano collection. Due to livestock‐induced failures in the energy supply, collections from Malaise trap locations in WI were unsuccessful. Collection permits were requested from relevant local authorities.

### 2.2 DNA Extraction

#### 2.2.1 Guano

A total of 298 single bat guano pellets and 33 samples with 5–10 pellets were extracted using the Quick‐DNA Fecal/Soil Microbe Kit (Zymo Research, Irvine, CA, USA) following the manufacturer's protocol. Extraction success and DNA quality were checked on a 1% agarose gel.

#### 2.2.2 Malaise Traps

Insect bulk samples (n=523) were size‐sorted in the collection ethanol into small (S, < 4 mm) and large (L, > 4 mm) size fractions by passing the sample through a 4 × 4 mm stainless‐steel mesh (wire diameter 0.5 mm). Depending on biomass, the resulting size fractions were transferred into 5 mL tubes (Eppendorf, Hamburg, Germany), 30 mL tubes (Nalgene, wide‐mouth bottle, Thermo Fisher Scientific, Waltham, MA, USA), or 40 mL or 100 mL Milling tubes (IKA MT 40 and MT 100, Staufen im Breisgau, Germany). Tubes were left open in an incubator (VWR, Radnor, PA, USA) at 50°C for up to 6 days to evaporate residual ethanol and dry the samples for homogenization. Based on the type of tube, samples were homogenized using either a Mixer mill (Retsch MM400, Haan, Germany) with metal beads (3 mm for 5 mL tubes/5 mm for Nalgene wide‐mouth bottle) for 5 min at a frequency of 30 s per cycle, or an IKA Tube Mill 100 (Staufen im Breisgau, Germany) for 3 min at 25,000 rpm. For each sample size fraction, approximately 20 mg of the resulting homogenized material was transferred into a 1.5 mL tube (Eppendorf, Hamburg, Germany) for subsequent DNA extraction using the DNeasy 96 Blood and Tissue Kit (Qiagen, Hilden, Germany) modified for insect bulk samples. Sample size fractions were incubated separately in 200 μL of ATL buffer and Proteinase K at 56°C overnight in a shaking incubator at 110 rpm (INCU Line ILS 6, VWR, Radnor, PA, USA). We combined 135 μL of the S fraction lysate and 15 μL of the L fraction lysate to reduce biomass bias and increase detection probability of small insects (Elbrecht et al. 2021), then followed the manufacturer's protocol for the rest of the extraction.

### 2.3 Amplicon Library Preparation

A two‐step PCR protocol using the PCR Multiplex Plus Kit (Qiagen, Hilden, Germany) was followed for amplicon library preparation (Thomas et al. 2024). All PCRs were conducted with an Applied Biosystems 2720 thermocycler. The initial PCR used primers fwhF2 (GGDACWGGWTGAACWGTWTAYCCHCC) (Vamos et al. 2017) and fol‐degen‐rev (TANACYTCNGGRTGNCCRAARAAYCA) (Yu et al. 2012), amplifying a 313 bp fragment of the mitochondrial cytochrome oxidase 1 (CO1) gene. PCR reaction mixtures comprised 12.5 μL Multiplex PCR Master Mix 2×, 0.2 μM of both forward and reverse primers, 11.9 μL of ddH_2_O, and 1 μL of DNA template. Cycling conditions included an initial denaturation at 95°C for 5 min, followed by 25 cycles of 30 s each at 95°C, 50°C, and 72°C, with a final extension step at 72°C for 5 min.

For PCR 2, the products from PCR 1 were used as template with Nextera tagging primers for indexing (Nextera, Illumina, San Diego, CA, USA). Reaction and cycling conditions were similar to PCR 1, but with 15 cycles instead of 25. PCR success was assessed using a 1% agarose gel. The products of PCR 2 were normalized using a SequalPrep normalization plate (Thermo Fisher Scientific, Waltham, MA, USA) according to the manufacturer's instructions, resulting in a final concentration of 25 ng per sample (20 μL). Subsequently, 10 μL aliquots of each sample were pooled together before undergoing two rounds of left‐sided size selection using magnetic beads (SPRIselect; Beckman Coulter, Brea, CA, USA) with a sample to bead ratio of 1:0.7 to eliminate any residual primer dimers. The pooled libraries were sent to Macrogen Europe (Amsterdam, The Netherlands) for sequencing on two HiSeq2500 sequencer (250 bp PE) (Illumina, San Diego, CA, USA), two lanes each. Raw sequence data for this project are available in the NCBI's SRA archive under accession number: PRJNA1119978.

### 2.4 Bioinformatic Processing

#### 2.4.1 Raw Data Processing

Demultiplexing was performed by Macrogen Europe. Primer pairs were removed using cutadapt 3.5 (Martin 2011) with the following settings: maximum error rate (e) 0.1 and minimum overlap (‐O) 20. Only sequences containing both the forward and reverse primers were kept for further analysis. In qiime2 (version 2023.2) (Bolyen et al. 2019), the sequences were truncated to 225 bp for the forward and reverse reads, then Dada2 (Callahan et al. 2016) was used for paired‐end read merging, quality filtering, chimera detection, and denoising. Sequences with ≥ 97% similarity were clustered into 811 Operational Taxonomic Units (OTUs) for the bat guano samples and 8486 OTUs for the Malaise trap samples using vsearch as implemented in qiime2. The R package “LULU” was used for further qualitative filtering, resulting in 811 OTUs and 8186 OTUs for the bat guano and Malaise trap samples, respectively. Taxonomic assignment of representative sequences was performed against the BOLD database using boldigger (Buchner and Leese 2020). The JAMP‐Pipeline option in boldigger was used to filter the result list.

In both the bat guano and Malaise trap datasets, all non‐arthropod OTUs were removed, as well as OTUs which were not assigned to species level or at a similarity of < 98%. All guano samples indicating additional bat species were removed from further analysis. OTUs assigned to the same species were merged. Sampling rounds were excluded if both types of samples were not collected and, for the guano data, if non‐*Plecotus* Chiroptera sequences were present. Removing these samples ensured that guano data reflected only the diet of our target *Plecotus* species. Reads from negative controls were subtracted from the samples on their respective plates, and OTUs with no remaining reads were dropped from the data set before converting it to presence‐absence. This resulted in a combined total of 3483 OTUs (henceforth “species”), of which 2382 were present either in the bat guano or Malaise traps nighttime samples (18.00–06.00). Pooled guano samples detected fewer taxa than the single pellets, however excluding the pooled samples did not change patterns found in the analysis. All guano samples were included in the analysis presented below.

#### 2.4.2 Statistical Analysis

Statistical analysis and data visualization were performed in R version 4.5.2 (R Core Team 2025). Community similarities were visualized using heat maps (metacoder v 0.3.8) (Foster et al. 2017) and upset plots (ggupset v 0.4.1) (Ahlmann‐Eltze 2020). Community dissimilarity between bat guano and Malaise trap catches was calculated within each sampling round and sampling area using the binary Jaccard index (vegan v 2.7–2) (Oksanen et al. 2013) and visualized as similarity (1‐dissimilarity). Detection rates throughout the sampling period were analyzed separately at the order, family, and species level using individual multinomial log‐linear models (nnet v 7.3–20) (Venables and Ripley 2013). Effects were predicted and plotted using ggeffects (v 2.3.1) (Lüdecke 2018). Temporal similarities between bat guano communities and Malaise trap catches were visualized from a generalized linear latent variable model (gllvm v 2.0.5) (Niku et al. 2020) using family binomial with probit link function and two latent variables. For more details, please see the R scripts at the link provided in the data availability statement.

## 3 Results

### 3.1 Confirmation of Hunting Grounds and Nest Sites

Camera traps installed at the roosts confirmed the presence of *Plecotus* sp. at the sites in Flamersheim, Berg, and the Eifel National Park. Although the camera trap at the Wildenburg roost malfunctioned, individual *Plecotus* bats were observed hanging freely under the roof truss during multiple site visits. Additionally, several individual *P. austriacus* were captured using mist nets at the “Weiher” site within the Eifel Nationalpark. Captured bats were tagged and tracked to the roosts in Foresters Lodge, further confirming the site as a hunting ground.

Molecular analysis of guano samples supported the camera findings. *P. auritus* was identified in samples from Eifel National Park and Wildenburg roosts, and *P. austriacus* was detected in Flamersheim and Berg. Further, metabarcoding of guano revealed the presence of additional bat species: *Pipistrellus pipistrellus* at the roost in Flamersheim, Wildenburg and the Eifel National Park; *Myotis myotis* at Berg and Wildenburg; and *Myotis daubentonii* at Flamersheim, indicating that these species may have occasionally roosted together with *Plecotus*. All guano samples indicating additional bat species were removed from further analysis.

Of the eleven AudioMoth devices deployed alongside Malaise traps, seven produced usable recordings. In total, 291,827 audio recordings were obtained, of which 1092 were identified as *Plecotus* calls. No *Plecotus* calls were recorded at the “Weiher” site in the Eifel National Park, however their presence was confirmed by mist‐net captures. Acoustic data and mist‐netting confirmed Wildenburg ZA & HW, Eifel National Park FH, WE, Berg SW & NG and Flamersheim BG as *Plecotus* hunting grounds (Table S1).

### 3.2 Diet of *Plecotus auritus* and *Plecotus austriacus*

In the curated guano dataset, the most diverse prey species were insects (292, 91.5% of assessed species; Figure S3). Additionally, 16 Arachnida (5.0%), 2 Isopoda (0.6%), and 4 Chilopoda (1.3%) species were found in guano samples of the two *Plecotus* species. The five most diverse orders recovered were Lepidoptera (133 species, 41.7%), Diptera (67 species, 21.0%), Coleoptera (24 species, 7.5%), Hemiptera (21 species, 6.6%), and Hymenoptera (20 species, 6.3%). All other orders were represented by < 10 species, together accounting for 16.9% of all insect species recovered.

**FIGURE 2:**
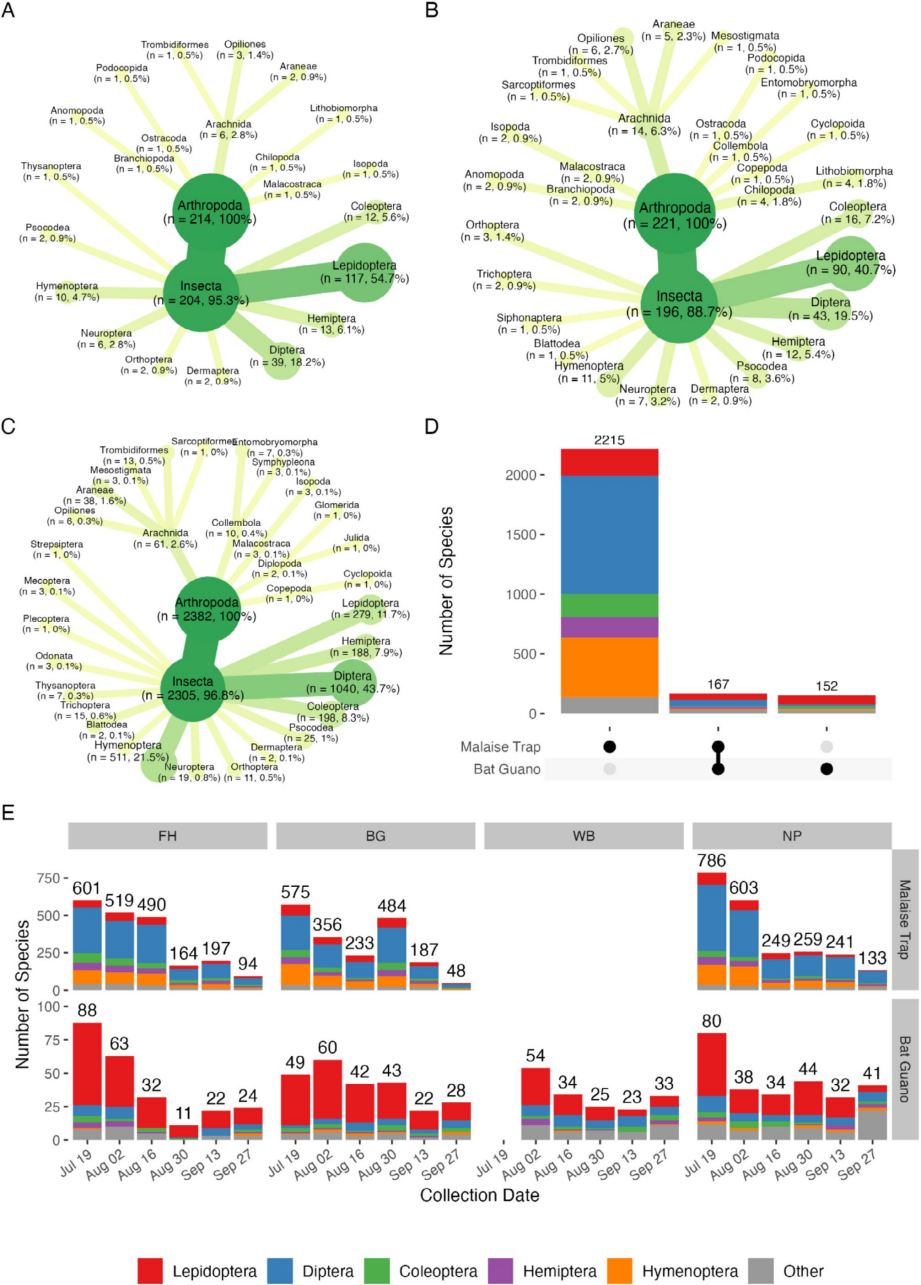
Number of insect taxa detected in guano samples of (A) *Plecotus austriacus* and (B) *Plecotus auritus* , or captured via (C) time‐controlled Malaise trap nighttime catches (18:00–06:00). (D) Overlap in species composition between guano‐derived and trap‐collected samples. (E) Temporal and spatial shifts in insect community composition across sampling sites and dates, for Malaise traps (upper) and guano samples (lower). *Plecotus austriacus* roosted at Flamersheim (FL) and Berg (BG). *Plecotus auritus* roosted at Wildenburg (WI) and Eifel National Park (NP).

The total number of arthropod species detected in bat guano was similar between the two *Plecotus* species, but the relative composition differed between species, colonies and sampling rounds (Figure 2). The diet of *P. austriacus* was dominated by Lepidoptera (Figure 2A), while *P. auritus* also consumed a comparatively high percentage of Arachnids, Diptera and Coeloperta (Figure 2B). Dietary diversity was most variable for the colony in Flamersheim, with the greatest number of different arthropod species found in guano from early July (Figure 2E; sampling round 1, 88 species) and the fewest in late August (round 4, 11 species), with a total of 186 species. For comparison, in Berg 131 arthropod species were detected overall, with the highest richness in late July (round 2, 60 species) and the lowest in early September (round 5, 22 species). The Wildenburg colony had a diet of 102 prey species, with the number of species detected per round varying between 54 (round 1, early July) and 23 (round 4, late August). In the Eifel National Park, 129 arthropod species were recovered, with a maximum of 80 (round 1, early July) and a minimum of 32 (round 5, early September).

The greatest insect diversity was recovered from bat guano early in the sampling period and the lowest in late August and early September. In all bat colonies, the number of detected Lepidoptera species decreased from July until September, while the number of Diptera and Coleoptera varied with no clear trend. Lepidoptera made up nearly 52% of detections in Wildenburg in late July, with a low of 21.7% in early September. In contrast, Lepidoptera never exceeded 20% of species detections in Flamersheim or NP, and ranged from 17.1%–27.6% in Berg. The lowest percentage of Lepidoptera species was 6.9% in Eifel National Park in late September. Notably, the greatest number of “Other” species, not belonging to the five most diverse orders, was recovered from the two *P. auritus* colonies in Wildenburg and Eifel National Park in late September (Figure 2E).

### 3.3 Prey Community/Circadian Rhythm of Flying Insects

In total, 3483 different arthropod species were identified in Malaise trap samples, of which 2382 were observed during the night (Figure 2C). Five orders made up, on average, 92.2% of the total number of species in the nighttime samples: Diptera (1040 species), Hymenoptera (511 species), Coleoptera (198 species), Lepidoptera (279 species) and Hemiptera (188 species). The total number of insect species collected with Malaise traps decreased over time at all locations, but the individual orders remain consistent in their proportions (Figure 2E). Highest richness was observed in the Eifel National Park, and the lowest from Wildenburg.

**FIGURE 3:**
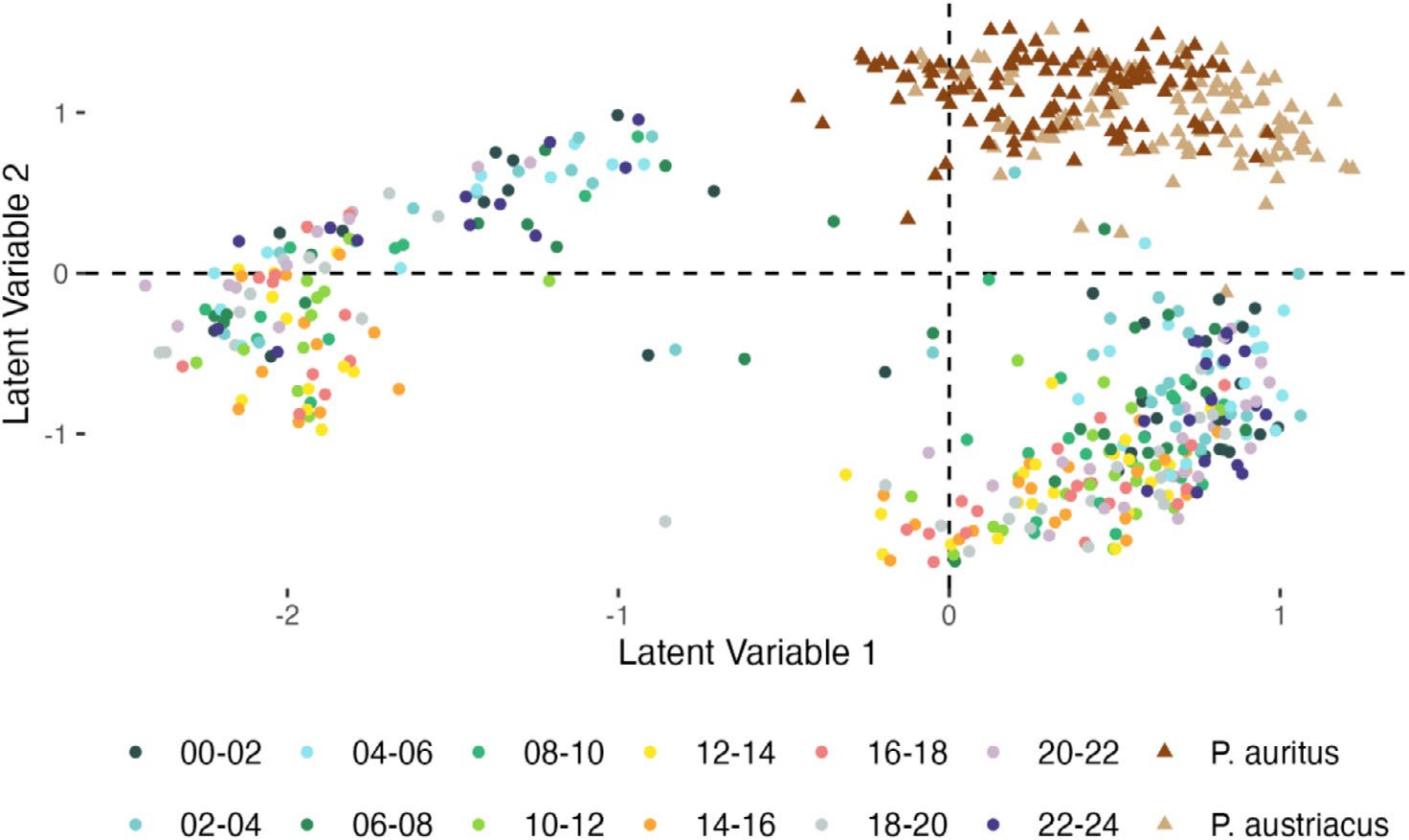
Arthropod species community composition recovered from bat guano (dark and light brown triangles; *Plecotus auritus and P. austriacus*, respectively) most closely resembled Malaise trap bulk arthropod samples (circles) from samples collected during the night (blue tones). Generalized linear latent variable model with two latent variables. Bluer tones represent nighttime samples and yellower tones daytime samples.

A model‐based ordination using generalized linear latent variable models (gllvm) revealed clear separation of bat guano samples from bulk insect samples. The potential prey community exhibited a pronounced cyclical pattern. Notably, samples from midday periods (12–16 h) were positioned furthest from guano samples, while nighttime bulk samples clustered closer to guano samples (Figure 3).

**FIGURE 4:**
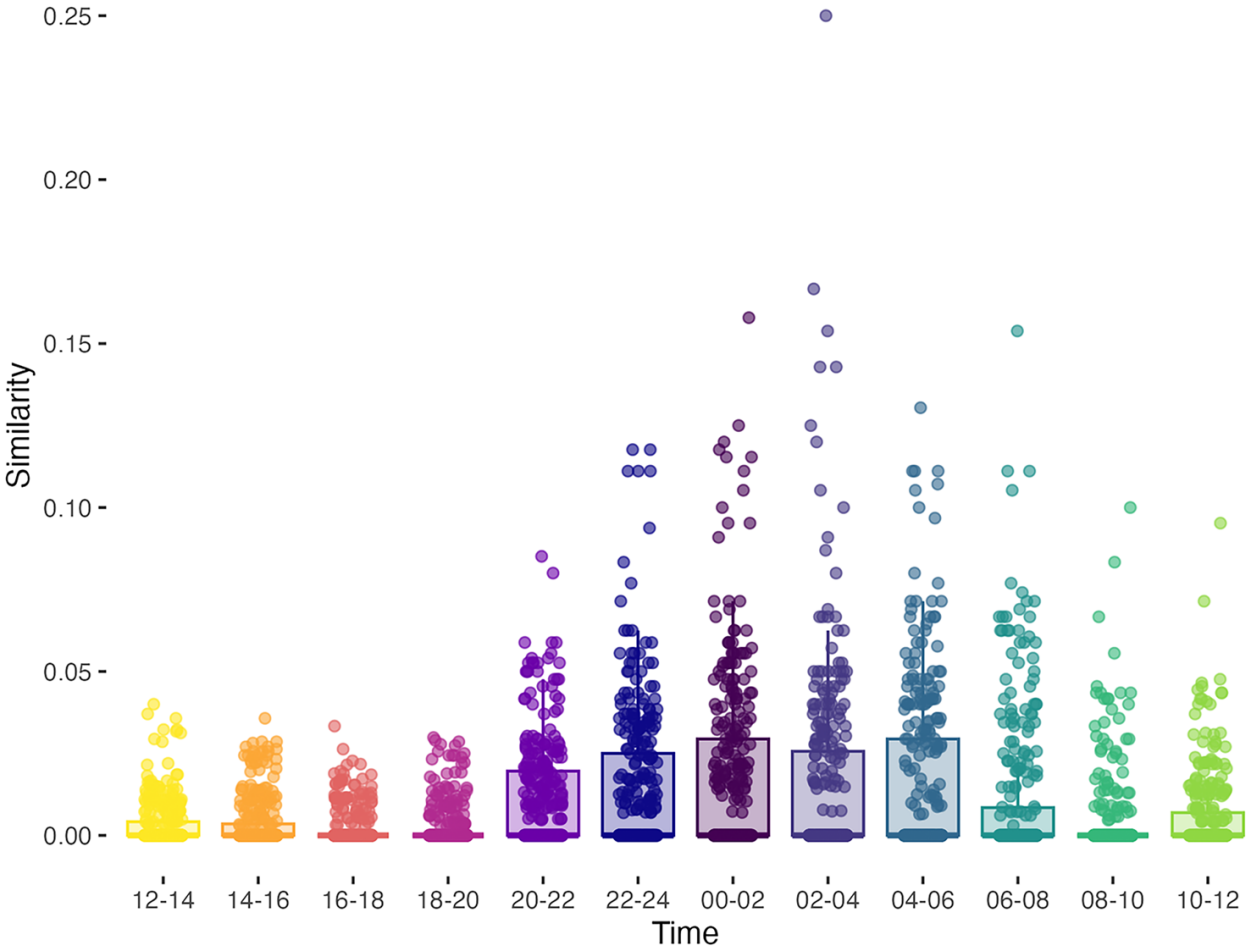
Arthropod community similarity (1‐Jaccard Dissimilarity Index) between guano samples and timed Malaise trap samples. Comparisons were calculated within each sampling round and sampling area using only OTUs identified to species level.

Guano arthropod communities were most similar to time‐controlled Malaise trap samples collected between 22:00 and 06:00 (Figure 4). Interestingly, this pattern was more pronounced for Lepidoptera but not Diptera prey and suggested a period of lower foraging activity around 22:00–24:00 for *P. auritus* but not *P. austriacus* (Figure S4).

### 3.4 Multinomial Predictions

**FIGURE 5:**
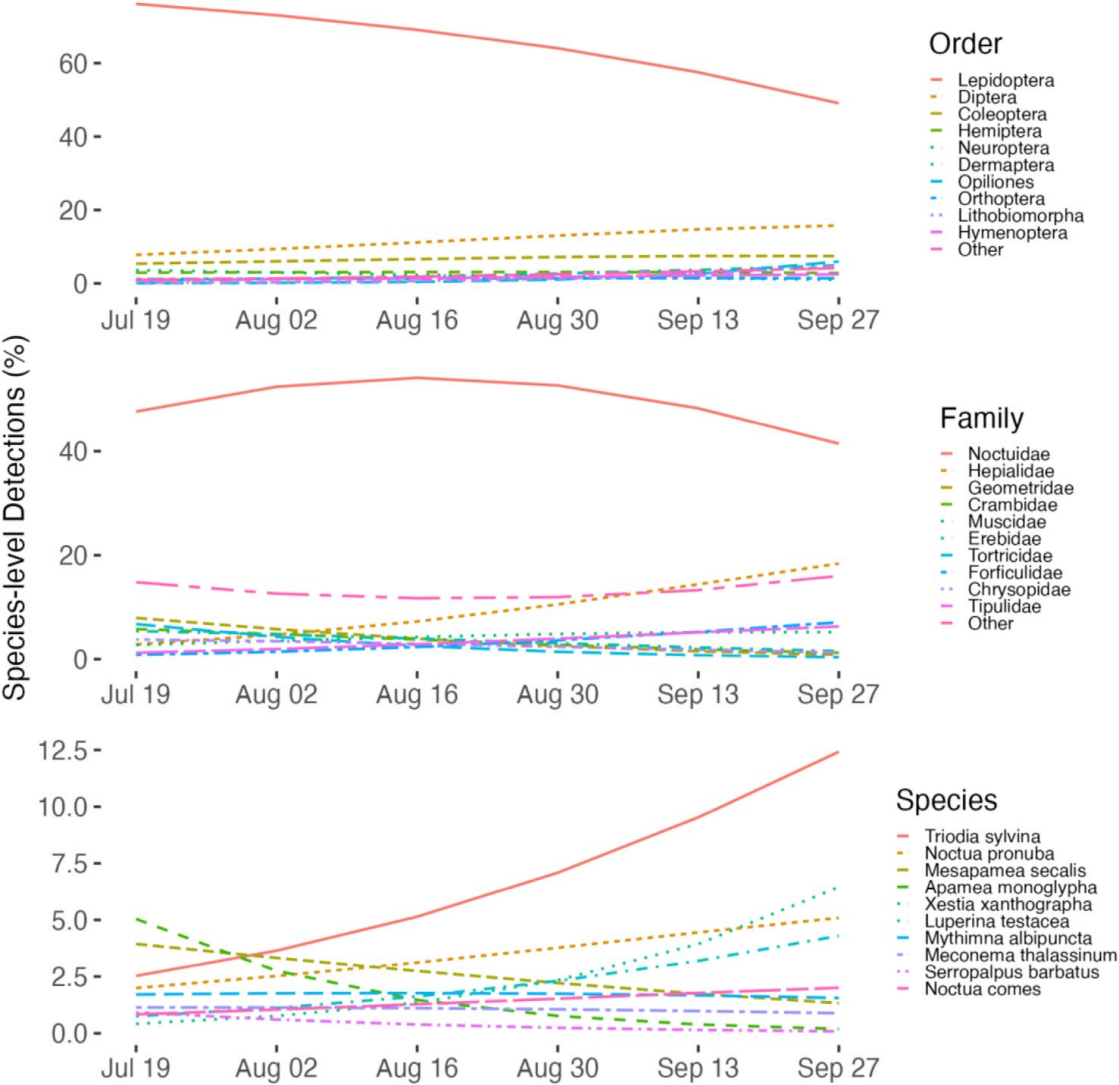
Species‐level detections from bat guano over time as percentage of total detections (proportion of samples in which a taxon was detected) by order (top) or family (center), and for the 10 most frequent species (bottom). The most frequent species are all Lepidoptera except for *Meconema thalassinum* (Orthoptera: Tettigoniidae) and *Serropalpus barbatus* (Coleoptera: Melandryidae).

The greatest number of species‐level detections from *Plecotus* bat guano were attributed to Lepidoptera (Figure 5). They made up the largest percentage of detections (76.1%–53.3%) regardless of the sampling round and were mainly noctuids, although the most frequently detected species was *Triodia sylvina* (Hepialidae). Toward autumn, Lepidoptera comprised a decreasing percentage of species detected while the number of Diptera species recovered from guano increased. Coleoptera species increased slightly over the sampling period, but did not exceed 8.3%. Araneae and Hemiptera fluctuated between 0.9%–2% and 3.4%–4.1%, respectively. Opiliones increased from < 1% to 9% of species in autumn. Similarly, the less frequent orders grouped as “Others” accounted for 6.2% of species detections in round 3, and increased to 14.5% in late September.

The arthropod families and species most frequently recovered during the night by Malaise traps were entirely different from those in bat guano. The potential prey community was dominated by Diptera (Figure S5; 50.5%–54.9%). In contrast to the seasonal differences detected from bat guano, similar proportions of Hymenoptera and Lepidoptera species were recovered throughout the sampling period and consistently represented < 20% of species‐level detections.

## 4 Discussion

Comparisons of bat molecular dietary data with temporal insect activity patterns obtained from time‐controlled Malaise traps enable assessment of their primary hunting times without disturbing the animals by attaching tracking devices or conducting close roost observations. Importantly, this combined approach can offer deeper insights into bat foraging strategies, allowing not only the identification of hunting periods but also direct comparison of bat activity with insect diversity, activity, and abundance. Our results underscore the utility of eDNA metabarcoding for inferring bat hunting strategies, highlight the limitations of single‐source data for drawing robust ecological conclusions, and demonstrate the utility of multimodal approaches for species interaction and ecosystem monitoring.

### 4.1 Inferring Bat Hunting Strategies Through Guano Analysis

Reconstructing food webs through fecal analysis is a well‐established method (Dickman and Huang 1988; Fukui and Agetsuma 2010; Joshi et al. 1997), but the advent of metabarcoding has unlocked a wealth of information on local prey species diversity from these “biodiversity capsules” (Boyer et al. 2015). Here we recovered more Lepidoptera species from *P. austriacus* guano, similar proportions of Diptera and Hemiptera species from the guano of both bats, and more arachnid species from *P. auritus* . These dietary patterns align with observations that *P. auritus* employs a gleaning strategy, capturing also prey from solid surfaces rather than during flight (Andriollo et al. 2021), while *P. austriacus* hunts mainly in open air. Consequently, nonflying prey species may represent a substantial component of the diet of *P. auritus* , while flying prey species dominate the diet of *P. austriacus*. The diet of *P. auritus* varied more over the 3‐month sampling period than did that of *P. austriacus* , which was dominated by Lepidoptera at all time points. This finding sheds light on the unresolved question to what extent annual variations in the overall arthropod community may be reflected in *Plecotus* guano (Hurpy et al. 2025).

### 4.2 Comparing Arthropod Communities From Bat Guano and Malaise Traps

While it has been suggested that detailed fecal analysis of apex predators may provide holistic representation of local prey communities (Shao et al. 2021), comparing eDNA metabarcoding of bat guano with metabarcoding time‐stamped Malaise trap bulk samples demonstrates that each method alone provides a unique view into local insect communities but neither is complete. A total of 167 arthropod species were identified in both Malaise trap samples and bat feces. However, an additional 2215 species were recovered exclusively from Malaise traps and 152 species uniquely from fecal samples. Thus integration of both sample types significantly increased the overall detection of arthropod diversity. Although bat guano itself is currently being used to assess insect communities (Curran et al. 2022; Liu, Si, et al. 2023; Tobisch et al. 2025), we recommend that fecal metabarcoding should be employed as a complementary approach alongside other monitoring techniques. Similar conclusions have been drawn in studies of other taxa, where observational methods combined with fecal metabarcoding substantially improved monitoring outcomes (Brun et al. 2022; Johnson et al. 2025).

Malaise trap catches were dominated by Diptera, but bat diets were characterized by a high proportion of Lepidoptera, followed by Diptera and taxa from less species‐rich groups such as Orthoptera and Arachnida, similar to previous diet studies (summarized in ClimBats, accessed 26 November 2025; (Froidevaux et al. 2023)). Differences between the potential prey community and actual consumption suggest trade‐offs between the energy cost of food capture, the nutritional value of particular types of prey, and the availability of preferred prey. Prey body size, and its corollary relative body size of the bat and potential prey items, have been frequently invoked as a key factor in bat foraging decisions (Divoll et al. 2022).

This pattern is reflected in the main prey species identified in our study. Among the ten species with the highest probability of detection in the guano samples, eight were larger moth species, ranging in size from 27 to 60 mm (Table S2). The two non‐lepidopteran taxa frequently detected in the guano samples have body sizes between 8 mm and 18 mm. *Meconema thalassinum* (Orthoptera: Tettigonidae) might have been captured in flight but was likely taken through gleaning behavior. Similarly, the frequently consumed coleopteran *Serropalpus barbatus* (Melandryidae) is also typically captured by gleaning. These findings support previous studies showing that larger body size increases likelihood of predation by aerial foraging, but plays a less important role during gleaning (Divoll et al. 2022).

It must be noted that in addition to influencing the likelihood of becoming prey, arthropod body size may also influence detection probability in eDNA‐based diet analyses (Liu, Burridge, et al. 2023; Schattanek et al. 2021). Although several authors have emphasized the use of relative read abundance (RRA) to estimate the proportional contribution of prey species (Deagle et al. 2019; Vesterinen et al. 2018), we argue that larger species contribute disproportionately more DNA to guano samples than smaller species, thereby increasing their likelihood of detection (Schattanek et al. 2021; Thuo et al. 2019). This issue is exacerbated by primer bias (Liu, Burridge, et al. 2023; Sickel et al. 2023) and incompleteness of databases, particularly for poorly cataloged “dark taxa” such as small Diptera and Hymenoptera. Additionally, many bats have a highly diverse diet, further limiting the applications of RRA (Littleford‐Colquhoun, Freeman, et al. 2022; Littleford‐Colquhoun, Sackett, et al. 2022).

Metabarcoding of feces is being applied to insect surveillance (Liu, Si, et al. 2023; McHale et al. 2025), however, our results indicate that monitoring endangered or invasive species cannot be achieved comprehensively through either guano metabarcoding or Malaise traps. Both datasets recovered endangered arthropod species (compared to the German Red List (Downloaded from https://www.rote‐liste‐zentrum.de/de/Download‐Wirbellose‐Tiere‐1875.html on 03.07.2024)) at our sampling locations (Table S3). Three endangered species were detected in bat guano, compared to 49 in Malaise trap samples, suggesting that Malaise trapping poses a substantially greater risk to endangered species than bat predation. While Malaise traps may directly threaten endangered populations, guano sampling is noninvasive and does not impose additional harm. Interestingly, analysis of bat guano revealed sequences of two widespread invasive species that were not detected in Malaise trap samples, while the traps contained 19 additional invasive arthropods (Table S4).

We believe our results provide a conservative picture of both the potential prey community and that portion which was actually consumed. The power of metabarcoding is directly linked to the completeness and quality of reference databases (Keck et al. 2023). Larger species are more likely to be represented, while smaller species are frequently absent, comprising a substantial fraction of so‐called “dark taxa” (Morinière et al. 2019). Dipterans, including night‐active mosquitos, account for a large share of those dark taxa (Chimeno et al. 2023), and as such we assume that metabarcoding results underestimate them. It is also important to note that endangered species are often underrepresented in reference databases, directly reducing their probability of detection. A similar limitation applies to invasive species, particularly when they have only recently been introduced into a habitat and are therefore absent from customized reference databases. Progress has been made in improving reference data quality, but taxa are missing or sequences incorrectly annotated even in well‐studied geographic regions (Chimeno et al. 2023).

### 4.3 eDNA Metabarcoding and Time‐Controlled Traps for Analysis of Species Interactions

Similarities between the Malaise trap catches and the guano samples provide a limited reflection of the individual preferred hunting times of these bat species, which leave their roost approximately 10–30 min after sunset and return approximately half an hour before sunrise (Starik et al. 2021). Differences can largely be explained by the limited effectiveness of Malaise traps for capturing one of *Plecotus'* preferred prey: moths are more reliably monitored using light traps (Busse et al. 2022). Indeed, we have frequently observed moths resting on the white mesh of Malaise traps; they may or may not enter the collecting bottle later, for example after being disturbed.

The dataset presented here also highlights how combining of methods can improve understanding of circadian and seasonal patterns in predator–prey responses. The observed shift in prey from July until October corresponds to known activity times of prey species. The calculated probability of detection for the eight most common species in the guano samples matches the typical activity time of those moths (Table S2), underscoring the potential for eDNA derived from guano samples in activity monitoring of larger moth species. This suggests that our method would also support predicting and assessing bats' responses to shifts in prey availability to climate shifts, an impact that has been largely neglected thus far (Kerth and Wolf 2025; Russo et al. 2025). It would be able to detect, for example, shifts between aerial capture and gleaning behavior, or complex interactions of temperature and day‐length on predator–prey relationships.

Combining fecal metabarcoding with other simultaneous collections of the arthropod community is rare. The only such studies we are aware of which combine predator fecal metabarcoding with direct sampling of the local arthropod community are limited to particular prey types in birds (Bookwalter et al. 2023; Rytkönen et al. 2019) and do not account for circadian patterns in arthropod availability. Incorporating temporal information on prey with dietary information from metabarcoding could improve the knowledge base used to inform conservation efforts, including regulating artificial light at night, turning off wind turbines during peak bat hunting periods, and managing vegetation to support diverse, stable food webs (Mariton et al. 2023). A comprehensive understanding of bat activity patterns is necessary for effective implementation of targeted conservation measures, such as mitigating light pollution during peak activity times or synchronizing wind turbine operation with bat resting periods (Mariton et al. 2023).

Taking the predator–prey relationship of bats and insects as a model for unpicking species interactions, we have demonstrated the great potential for multimodal monitoring built on this framework.

Our results support the utility of eDNA metabarcoding of feces for reconstructing food webs and highlight the potential for combining multiple methods to improve inferences related to bat behavior and ecological networks. In contrast to traditional diet studies, which usually provide a static snapshot of predator–prey interactions, the dataset presented here combines time‐resolved guano metabarcoding with synchronized insect activity data. This enables dynamic modeling of trophic interactions over time, rather than simply providing a list of what was eaten without temporal or ecological context. This fine‐scale approach to predator–prey relationships can capture fluctuations on fine temporal and potentially spatial scales, offering a more realistic and responsive view of food‐web dynamics in changing environments and improving the basis for conservation and management decisions.

## 5 Conclusions

Understanding bat foraging strategies requires not only dietary and activity data but also detailed information on insect phenology and traits. While molecular dietary analyses and time‐controlled Malaise traps provide powerful insights into bat hunting times and their alignment with insect activity peaks, the full potential of such approaches can only be realized when embedded within comprehensive trait databases. The foundation of such a database has been established for bats (ClimBats, Froidevaux et al. 2023), but an open, user‐friendly platform is lacking for insects.

The recently proposed Global Repository of Insect Traits (GRIT) highlights the urgent need for a centralized, FAIR‐accessible platform that compiles insect traits across taxa and regions (Cardoso et al. 2025). By integrating data on morphology, life history, physiology, behavior, and phenology, trait databases are enabling researchers to link predator activity patterns with prey availability at unprecedented scales. For bat–insect interaction studies, such a repository would allow systematic analyses of how bat foraging flexibility responds to insect seasonal dynamics, temperature thresholds, and habitat associations.

By combining trait databases with molecular and temporal monitoring approaches, multidimensional insights can be generated: identifying mismatches between bat hunting activity and insect abundance peaks, predicting how climate change may disrupt synchrony, and assessing cascading effects on food‐web stability. Thus, the integration of multimodal sampling with GRIT‐like resources into bat–insect research represents a crucial step toward advancing both ecological understanding and conservation planning.

## Author Contributions

**Tamara R. Hartke:** data curation (equal), formal analysis (lead), visualization (lead), writing – original draft (equal), writing – review and editing (equal). **Manus Wittenhorst:** formal analysis (supporting), visualization (supporting), writing – original draft (supporting), writing – review and editing (supporting). **Martin Koch:** conceptualization (equal), formal analysis (equal), investigation (supporting), writing – original draft (supporting), writing – review and editing (supporting). **Christoph Scherber:** formal analysis (equal), methodology (supporting), project administration (supporting), resources (equal), writing – original draft (supporting), writing – review and editing (equal). **Sönke Twietmeyer:** formal analysis (supporting), investigation (supporting), methodology (supporting), resources (supporting), writing – original draft (supporting), writing – review and editing (equal). **Sarah J. Bourlat:** methodology (supporting), writing – original draft (supporting), writing – review and editing (equal). **Michel Böhme:** investigation (equal), methodology (supporting), writing – original draft (supporting), writing – review and editing (equal). **Ameli Kirse:** conceptualization (lead), data curation (equal), formal analysis (equal), investigation (lead), methodology (lead), project administration (lead), supervision (lead), validation (equal), visualization (supporting), writing – original draft (equal), writing – review and editing (equal).

## Funding

This work is an outcome of the AMMOD project funded by the German Federal Ministry of Education and Research (grant number 01LC1903A).

## Conflicts of Interest

The authors declare no conflicts of interest.

## Supporting information


**Table S1:** Locations of sampling sites.
**Table S2:** Adult activity periods of the ten species most commonly found in guano samples, based on GBIF observation records for Germany.
**Table S3:** Endangered species recovered by metabarcoding, in alphabetical order by Genus. RL = Red List category: 1 = critically endangered; 2 = endangered; 3 = vulnerable.
**Table S4:** Invasive species (DAISIE and/or NOBANIS lists) recovered by metabarcoding, in alphabetical order by Genus.
**Figure S1:** Infrared camera images showing two *Plecotus* sp. individuals roosting on the ceiling of the nave in the Evangelical Church of Flamersheim.
**Figure S2:** Number of insect taxa detected in guano samples from both bat species combined.
**Figure S3:** Arthropod community similarity (1‐Jaccard Dissimilarity Index) between guano samples and timed Malaise trap samples. Comparisons were calculated within each sampling round and sampling area using only OTUs identified to species level. Top: subset of Lepidoptera (left) and Diptera (right) species only. Middle: subset of guano samples from *P. austriacus* (left) and *P. auritus* (right). Bottom: all comparisons by date considering all time points (left) and only evening and nighttime samples (right). Note different scales on the y‐axis.
**Figure S4:** Species‐level detections from Malaise traps over time as percent of total detections by order (top) or family (center), and for the 10 most frequent species (bottom).
**Figure S5:**. The AMMOD Multisampler installed in the field, and a detailed view of the rotation plate with the screwed‑in collection bottles. Position 13 remains empty to allow for breaks during sampling and to give insects the opportunity to escape during these pauses. For this study, positions 1–12 were equipped with collection bottles. The rotation plate turned every 2 h, skipping position 13, enabling continuous monitoring. Each bottle was positioned under the collection head at the exact same time of day across the 14‐day sampling period.

## Data Availability

Raw sequence data for this project are available in the NCBI's SRA archive under accession number: PRJNA1119978. The data analysis project (DOI 10.5281/zenodo.17831245).
