# Supplementary material for: Combining Time‐Stamped Insect Sampling With eDNA‐Metabarcoding of Guano to Reconstruct Community Interactions
Source: Ecol Evol. 2026 Apr 17;16(4):e73509. doi: 10.1002/ece3.73509 (PMC13090109; doi:10.1002/ece3.73509)
Supplement: Supplementary file 1 — Table S1: Locations of sampling sites. Table S2: Adult activity periods of the ten species most commonly found in guano samples, based on GBIF observation records for Germany. Table S3: Endangered species recovered by metabarcoding, in alphabetical order by Genus. RL = Red List category: 1 = critically endangered; 2 = endangered; 3 = vulnerable. Table S4: Invasive species (DAISIE and/or NOBANIS lists) recovered by metabarcoding, in alphabetical order by Genus. Figure S1: Infrared camera images showing two Plecotus sp. individuals roosting on the ceiling of the nave in the Evangelical Church of Flamersheim. Figure S2: Number of insect taxa detected in guano samples from both bat species combined. Figure S3: Arthropod community similarity (1‐Jaccard Dissimilarity Index) between guano samples and timed Malaise trap samples. Comparisons were calculated within each sampling round and sampling area using only OTUs identified to species level. Top: subset of Lepidoptera (left) and Diptera (right) species only. Middle: subset of guano samples from P. austriacus (left) and P. auritus (right). Bottom: all comparisons by date considering all time points (left) and only evening and nighttime samples (right). Note different scales on the y‐axis. Figure S4: Species‐level detections from Malaise traps over time as percent of total detections by order (top) or family (center), and for the 10 most frequent species (bottom). Figure S5:. The AMMOD Multisampler installed in the field, and a detailed view of the rotation plate with the screwed‑in collection bottles. Position 13 remains empty to allow for breaks during sampling and to give insects the opportunity to escape during these pauses. For this study, positions 1–12 were equipped with collection bottles. The rotation plate turned every 2 h, skipping position 13, enabling continuous monitoring. Each bottle was positioned under the collection head at the exact same time of day across the 14‐day sampling period. [file ECE3-16-e73509-s001.docx]

Supplementary Material for the manuscript:

**Combining time-stamped insect sampling with eDNA-metabarcoding of guano to reconstruct community interactions**

Hartke et al. 2026; Ecology and Evolution

Corresponding author: Ameli Kirse [a.kirse@leibniz-lib.de](mailto:a.kirse@leibniz-lib.de)

***Table S1*** - Locations of sampling sites


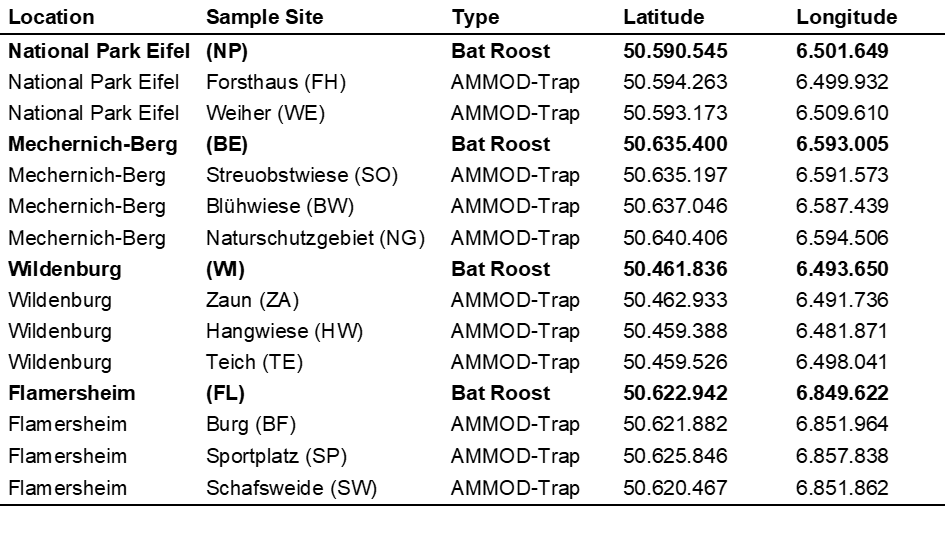


***Table S2* -** Adult activity periods of the ten species most commonly found in guano samples, based on GBIF observation records for Germany.**
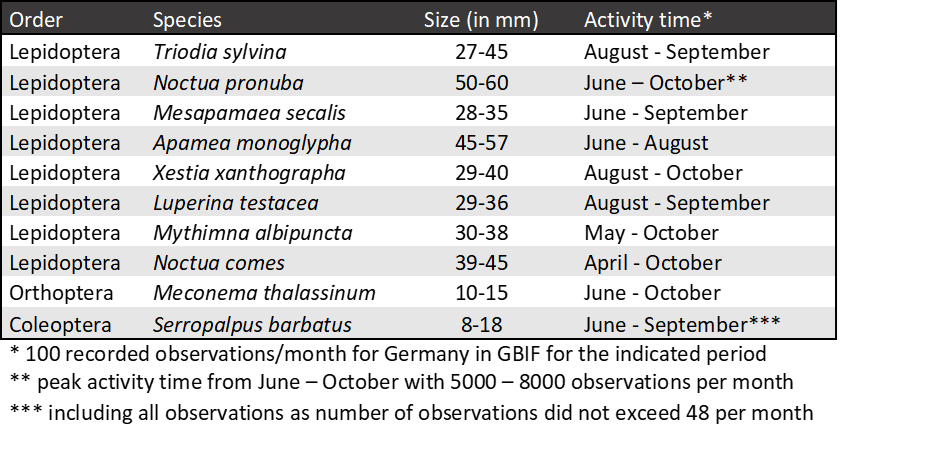
**

***Table S3*** Endangered species recovered by metabarcoding, in alphabetical order by Genus. RL = Red List category: 1 = critically endangered; 2 = endangered; 3 = vulnerable.

| **Species** | **Class** | **Order** | **Family** | **RL** | **Source** |
| --- | --- | --- | --- | --- | --- |
| *Aphanus rolandri* | Insecta | Hemiptera | Rhyparochromidae | 3 | Malaise |
| *Aporophyla lueneburgensis* | Insecta | Lepidoptera | Noctuidae | 1 | guano |
| *Arboridia pusilla* | Insecta | Hemiptera | Cicadellidae | 3 | Malaise |
| *Bagous lutulentus* | Insecta | Coleoptera | Curculionidae | 3 | guano |
| *Bicellaria intermedia* | Insecta | Diptera | Hybotidae | 3 | Malaise |
| *Bruchus pisorum* | Insecta | Coleoptera | Chrysomelidae | 1 | Malaise |
| *Coelioxys afra* | Insecta | Hymenoptera | Megachilidae | 3 | Malaise |
| *Coenagrion hylas* | Insecta | Odonata | Coenagrionidae | 0 | Malaise |
| *Cryptocephalus macellus* | Insecta | Coleoptera | Chrysomelidae | 3 | Malaise |
| *Dibolia timida* | Insecta | Coleoptera | Chrysomelidae | 3 | Malaise |
| *Dolichopus grandicornis* | Insecta | Diptera | Dolichopodidae | 0 | Malaise |
| *Drapetis arcuata* | Insecta | Diptera | Hybotidae | 3 | Malaise |
| *Empis vitripennis* | Insecta | Diptera | Empididae | 3 | Malaise |
| *Euglenes oculatus* | Insecta | Coleoptera | Aderidae | 3 | Malaise |
| *Euscelis ohausi* | Insecta | Hemiptera | Cicadellidae | 3 | Malaise |
| *Gymnetron melanarium* | Insecta | Coleoptera | Curculionidae | 3 | Malaise |
| *Halictus quadricinctus* | Insecta | Hymenoptera | Halictidae | 3 | Malaise |
| *Hardya tenuis* | Insecta | Hemiptera | Cicadellidae | 3 | Malaise |
| *Hercostomus nigripennis* | Insecta | Diptera | Dolichopodidae | 3 | Malaise |
| *Hercostomus rusticus* | Insecta | Diptera | Dolichopodidae | 3 | Malaise |
| *Hilara albitarsis* | Insecta | Diptera | Empididae | 3 | Malaise |
| *Himacerus boops* | Insecta | Hemiptera | Nabidae | 3 | Malaise |
| *Lasioglossum costulatum* | Insecta | Hymenoptera | Halictidae | 3 | Malaise |
| *Lasioglossum pauperatum* | Insecta | Hymenoptera | Halictidae | 2 | Malaise |
| *Lithophane semibrunnea* | Insecta | Lepidoptera | Noctuidae | 3 | Malaise |
| *Longitarsus ochroleucus* | Insecta | Coleoptera | Chrysomelidae | 3 | guano |
| *Medetera abstrusa* | Insecta | Diptera | Dolichopodidae | 3 | Malaise |
| *Medetera belgica* | Insecta | Diptera | Dolichopodidae | 3 | Malaise |
| *Medetera borealis* | Insecta | Diptera | Dolichopodidae | 1 | Malaise |
| *Medetera lorea* | Insecta | Diptera | Dolichopodidae | 1 | Malaise |
| *Medetera signaticornis* | Insecta | Diptera | Dolichopodidae | 3 | Malaise |
| *Medetera tristis* | Insecta | Diptera | Dolichopodidae | 3 | Malaise |
| *Megachile pilidens* | Insecta | Hymenoptera | Megachilidae | 3 | Malaise |
| *Myrmica schencki* | Insecta | Hymenoptera | Formicidae | 3 | Malaise |
| *Myrmica specioides* | Insecta | Hymenoptera | Formicidae | 3 | Malaise |
| *Neurigona suturalis* | Insecta | Diptera | Dolichopodidae | 3 | Malaise |
| *Oedalea flavipes* | Insecta | Diptera | Hybotidae | 3 | Malaise |
| *Otiorhynchus ligneus* | Insecta | Coleoptera | Curculionidae | 3 | Malaise |
| *Perapion affine* | Insecta | Coleoptera | Brentidae | 3 | Malaise |
| *Phyllotreta scheuchi* | Insecta | Coleoptera | Chrysomelidae | 2 | Malaise |
| *Plagiognathus fulvipennis* | Insecta | Hemiptera | Miridae | 3 | Malaise |
| *Platypalpus optivus* | Insecta | Diptera | Hybotidae | 3 | Malaise |
| *Platypalpus pygialis* | Insecta | Diptera | Hybotidae | 3 | Malaise |
| *Ponera coarctata* | Insecta | Hymenoptera | Formicidae | 3 | Malaise |
| *Pyrausta nigrata* | Insecta | Lepidoptera | Crambidae | 3 | Malaise |
| *Rhaphium appendiculatum* | Insecta | Diptera | Dolichopodidae | 3 | Malaise |
| *Rhytistylus proceps* | Insecta | Hemiptera | Cicadellidae | 3 | Malaise |
| *Sitona waterhousei* | Insecta | Coleoptera | Curculionidae | 3 | Malaise |
| *Tettigometra atra* | Insecta | Hemiptera | Tettigometridae | 2 | Malaise |
| *Thymelicus acteon* | Insecta | Lepidoptera | Hesperiidae | 3 | Malaise |
| *Tuponia hippophaes* | Insecta | Hemiptera | Miridae | 1 | Malaise |
| *Zygina rubrovittata* | Insecta | Hemiptera | Cicadellidae | 3 | Malaise |

***Table S4*** Invasive species (DAISIE and/or NOBANIS lists) recovered by metabarcoding, in alphabetical order by Genus.

| **Species** | **Class** | **Order** | **Family** | **Source** |
| --- | --- | --- | --- | --- |
| *Aphis gossypii* | Insecta | Hemiptera | Aphididae | guano |
| *Arhopalus rusticus* | Insecta | Coleoptera | Cerambycidae | guano |
| *Attagenus pellio* | Insecta | Coleoptera | Dermestidae | guano |
| *Cameraria ohridella* | Insecta | Lepidoptera | Gracillariidae | guano |
| *Cydalima perspectalis* | Insecta | Lepidoptera | Crambidae | guano |
| *Cydia splendana* | Insecta | Lepidoptera | Tortricidae | guano |
| *Diprion pini* | Insecta | Hymenoptera | Diprionidae | guano |
| *Dorypteryx domestica* | Insecta | Psocodea | Psyllipsocidae | guano |
| *Drosophila suzukii* | Insecta | Diptera | Drosophilidae | guano |
| *Eulachnus agilis* | Insecta | Hemiptera | Aphididae | guano |
| *Forficula auricularia* | Insecta | Dermaptera | Forficulidae | guano |
| *Gastrodes grossipes* | Insecta | Hemiptera | Rhyparochromidae | guano |
| *Liposcelis rufa* | Insecta | Psocodea | Liposcelididae | guano |
| *Orientus ishidae* | Insecta | Hemiptera | Cicadellidae | guano |
| *Philaenus spumarius* | Insecta | Hemiptera | Aphrophoridae | guano |
| *Ptinus fur* | Insecta | Coleoptera | Ptinidae | guano |
| *Rhagoletis completa* | Insecta | Diptera | Tephritidae | guano |
| *Strophosoma melanogrammum* | Insecta | Coleoptera | Curculionidae | guano |
| *Acleris variegana* | Insecta | Lepidoptera | Tortricidae | Malaise |
| *Aedes vexans* | Insecta | Diptera | Culicidae | Malaise |
| *Aleochara bipustulata* | Insecta | Coleoptera | Staphylinidae | Malaise |
| *Aleochara sparsa* | Insecta | Coleoptera | Staphylinidae | Malaise |
| *Aleyrodes proletella* | Insecta | Hemiptera | Aleyrodidae | Malaise |
| *Ametastegia pallipes* | Insecta | Hymenoptera | Tenthredinidae | Malaise |
| *Amischa analis* | Insecta | Coleoptera | Staphylinidae | Malaise |
| *Amphiareus obscuriceps* | Insecta | Hemiptera | Anthocoridae | Malaise |
| *Anagrus atomus* | Insecta | Hymenoptera | Mymaridae | Malaise |
| *Anotylus nitidulus* | Insecta | Coleoptera | Staphylinidae | Malaise |
| *Aphis craccivora* | Insecta | Hemiptera | Aphididae | Malaise |
| *Aphis cytisorum* | Insecta | Hemiptera | Aphididae | Malaise |
| *Aphis pomi* | Insecta | Hemiptera | Aphididae | Malaise |
| *Apis mellifera* | Insecta | Hymenoptera | Apidae | Malaise |
| *Appendiseta robiniae* | Insecta | Hemiptera | Aphididae | Malaise |
| *Aproaerema anthyllidella* | Insecta | Lepidoptera | Gelechiidae | Malaise |
| *Argiope bruennichi* | Arachnida | Araneae | Araneidae | Malaise |
| *Athalia rosae* | Insecta | Hymenoptera | Tenthredinidae | Malaise |
| *Atheta atramentaria* | Insecta | Coleoptera | Staphylinidae | Malaise |
| *Atheta fungi* | Insecta | Coleoptera | Staphylinidae | Malaise |
| *Atheta oblita* | Insecta | Coleoptera | Staphylinidae | Malaise |
| *Atomaria fuscata* | Insecta | Coleoptera | Cryptophagidae | Malaise |
| *Atomaria nitidula* | Insecta | Coleoptera | Cryptophagidae | Malaise |
| *Bemisia afer* | Insecta | Hemiptera | Aleyrodidae | Malaise |
| *Blastobasis phycidella* | Insecta | Lepidoptera | Blastobasidae | Malaise |
| *Bombus hortorum* | Insecta | Hymenoptera | Apidae | Malaise |
| *Bombus lucorum* | Insecta | Hymenoptera | Apidae | Malaise |
| *Bruchidius varius* | Insecta | Coleoptera | Chrysomelidae | Malaise |
| *Bruchus pisorum* | Insecta | Coleoptera | Chrysomelidae | Malaise |
| *Bruchus rufimanus* | Insecta | Coleoptera | Chrysomelidae | Malaise |
| *Cameraria ohridella* | Insecta | Lepidoptera | Gracillariidae | Malaise |
| *Carpelimus corticinus* | Insecta | Coleoptera | Staphylinidae | Malaise |
| *Cartodere bifasciata* | Insecta | Coleoptera | Latridiidae | Malaise |
| *Cartodere nodifer* | Insecta | Coleoptera | Latridiidae | Malaise |
| *Chaetocnema hortensis* | Insecta | Coleoptera | Chrysomelidae | Malaise |
| *Chrysoesthia sexguttella* | Insecta | Lepidoptera | Gelechiidae | Malaise |
| *Coccophagus gossypariae* | Insecta | Hymenoptera | Aphelinidae | Malaise |
| *Coleophora versurella* | Insecta | Lepidoptera | Coleophoridae | Malaise |
| *Copidosoma floridanum* | Insecta | Hymenoptera | Encyrtidae | Malaise |
| *Corticaria elongata* | Insecta | Coleoptera | Latridiidae | Malaise |
| *Corticaria serrata* | Insecta | Coleoptera | Latridiidae | Malaise |
| *Cryptolestes ferrugineus* | Insecta | Coleoptera | Laemophloeidae | Malaise |
| *Cryptophagus dentatus* | Insecta | Coleoptera | Cryptophagidae | Malaise |
| *Crypturgus subcribrosus* | Insecta | Coleoptera | Curculionidae | Malaise |
| *Cydia splendana* | Insecta | Lepidoptera | Tortricidae | Malaise |
| *Cypha pulicaria* | Insecta | Coleoptera | Staphylinidae | Malaise |
| *Didea intermedia* | Insecta | Diptera | Syrphidae | Malaise |
| *Dorypteryx domestica* | Insecta | Psocodea | Psyllipsocidae | Malaise |
| *Drosophila suzukii* | Insecta | Diptera | Drosophilidae | Malaise |
| *Dysaphis devecta* | Insecta | Hemiptera | Aphididae | Malaise |
| *Ectobius lapponicus* | Insecta | Blattodea | Ectobiidae | Malaise |
| *Ectopsocus briggsi* | Insecta | Psocodea | Ectopsocidae | Malaise |
| *Ectopsocus meridionalis* | Insecta | Psocodea | Ectopsocidae | Malaise |
| *Emmelina monodactyla* | Insecta | Lepidoptera | Pterophoridae | Malaise |
| *Empicoris rubromaculatus* | Insecta | Hemiptera | Reduviidae | Malaise |
| *Enderleinella obsoleta* | Insecta | Psocodea | Paracaeciliidae | Malaise |
| *Epitrix pubescens* | Insecta | Coleoptera | Chrysomelidae | Malaise |
| *Eratophyes amasiella* | Insecta | Lepidoptera | Oecophoridae | Malaise |
| *Eulachnus agilis* | Insecta | Hemiptera | Aphididae | Malaise |
| *Eumerus funeralis* | Insecta | Diptera | Syrphidae | Malaise |
| *Eupteryx decemnotata* | Insecta | Hemiptera | Cicadellidae | Malaise |
| *Eupteryx melissae* | Insecta | Hemiptera | Cicadellidae | Malaise |
| *Forficula auricularia* | Insecta | Dermaptera | Forficulidae | Malaise |
| *Glischrochilus quadrisignatus* | Insecta | Coleoptera | Nitidulidae | Malaise |
| *Grypotes puncticollis* | Insecta | Hemiptera | Cicadellidae | Malaise |
| *Harmonia axyridis* | Insecta | Coleoptera | Coccinellidae | Malaise |
| *Hyadaphis passerinii* | Insecta | Hemiptera | Aphididae | Malaise |
| *Hypera postica* | Insecta | Coleoptera | Curculionidae | Malaise |
| *Hypoponera punctatissima* | Insecta | Hymenoptera | Formicidae | Malaise |
| *Impatientinum asiaticum* | Insecta | Hemiptera | Aphididae | Malaise |
| *Lasioglossum malachurum* | Insecta | Hymenoptera | Halictidae | Malaise |
| *Lasius alienus* | Insecta | Hymenoptera | Formicidae | Malaise |
| *Lasius flavus* | Insecta | Hymenoptera | Formicidae | Malaise |
| *Lasius fuliginosus* | Insecta | Hymenoptera | Formicidae | Malaise |
| *Lepidocyrtus cyaneus* | Collembola | Entomobryomorpha | Entomobryidae | Malaise |
| *Leucoptera malifoliella* | Insecta | Lepidoptera | Lyonetiidae | Malaise |
| *Liriomyza bryoniae* | Insecta | Diptera | Agromyzidae | Malaise |
| *Lyctocoris campestris* | Insecta | Hemiptera | Lyctocoridae | Malaise |
| *Megaselia gregaria* | Insecta | Diptera | Phoridae | Malaise |
| *Mermessus trilobatus* | Arachnida | Araneae | Linyphiidae | Malaise |
| *Myzus ascalonicus* | Insecta | Hemiptera | Aphididae | Malaise |
| *Myzus cerasi* | Insecta | Hemiptera | Aphididae | Malaise |
| *Nearctaphis bakeri* | Insecta | Hemiptera | Aphididae | Malaise |
| *Oligota pusillima* | Insecta | Coleoptera | Staphylinidae | Malaise |
| *Orientus ishidae* | Insecta | Hemiptera | Cicadellidae | Malaise |
| *Oxycarenus lavaterae* | Insecta | Hemiptera | Oxycarenidae | Malaise |
| *Oxypoda haemorrhoa* | Insecta | Coleoptera | Staphylinidae | Malaise |
| *Pararge aegeria* | Insecta | Lepidoptera | Nymphalidae | Malaise |
| *Peripsocus milleri* | Insecta | Psocodea | Peripsocidae | Malaise |
| *Peripsocus parvulus* | Insecta | Psocodea | Peripsocidae | Malaise |
| *Phasia barbifrons* | Insecta | Diptera | Tachinidae | Malaise |
| *Philaenus spumarius* | Insecta | Hemiptera | Aphrophoridae | Malaise |
| *Philonthus concinnus* | Insecta | Coleoptera | Staphylinidae | Malaise |
| *Philonthus quisquiliarius* | Insecta | Coleoptera | Staphylinidae | Malaise |
| *Phyllonorycter issikii* | Insecta | Lepidoptera | Gracillariidae | Malaise |
| *Phyllonorycter leucographella* | Insecta | Lepidoptera | Gracillariidae | Malaise |
| *Phyllonorycter messaniella* | Insecta | Lepidoptera | Gracillariidae | Malaise |
| *Pieris rapae* | Insecta | Lepidoptera | Pieridae | Malaise |
| *Plutella xylostella* | Insecta | Lepidoptera | Plutellidae | Malaise |
| *Ponera coarctata* | Insecta | Hymenoptera | Formicidae | Malaise |
| *Pseudeuophrys lanigera* | Arachnida | Araneae | Salticidae | Malaise |
| *Psylliodes chrysocephalus* | Insecta | Coleoptera | Chrysomelidae | Malaise |
| *Rhyzobius lophanthae* | Insecta | Coleoptera | Coccinellidae | Malaise |
| *Sericoderus lateralis* | Insecta | Coleoptera | Corylophidae | Malaise |
| *Sitona discoideus* | Insecta | Coleoptera | Curculionidae | Malaise |
| *Stegobium paniceum* | Insecta | Coleoptera | Ptinidae | Malaise |
| *Stelidota geminata* | Insecta | Coleoptera | Nitidulidae | Malaise |
| *Stictoleptura rubra* | Insecta | Coleoptera | Cerambycidae | Malaise |
| *Stigmella atricapitella* | Insecta | Lepidoptera | Nepticulidae | Malaise |
| *Stigmella aurella* | Insecta | Lepidoptera | Nepticulidae | Malaise |
| *Strophosoma melanogrammum* | Insecta | Coleoptera | Curculionidae | Malaise |
| *Sturmia bella* | Insecta | Diptera | Tachinidae | Malaise |
| *Tachyporus nitidulus* | Insecta | Coleoptera | Staphylinidae | Malaise |
| *Thrips tabaci* | Insecta | Thysanoptera | Thripidae | Malaise |
| *Tingis cardui* | Insecta | Hemiptera | Tingidae | Malaise |
| *Trialeurodes vaporariorum* | Insecta | Hemiptera | Aleyrodidae | Malaise |
| *Tribolium castaneum* | Insecta | Coleoptera | Tenebrionidae | Malaise |
| *Trichogramma brassicae* | Insecta | Hymenoptera | Trichogrammatidae | Malaise |
| *Trichopsocus dalii* | Insecta | Psocodea | Trichopsocidae | Malaise |
| *Tuponia hippophaes* | Insecta | Hemiptera | Miridae | Malaise |
| *Tychius picirostris* | Insecta | Coleoptera | Curculionidae | Malaise |
| *Vespula germanica* | Insecta | Hymenoptera | Vespidae | Malaise |
| *Vespula vulgaris* | Insecta | Hymenoptera | Vespidae | Malaise |
| *Xantholinus linearis* | Insecta | Coleoptera | Staphylinidae | Malaise |
| *Xantholinus longiventris* | Insecta | Coleoptera | Staphylinidae | Malaise |
| *Xyleborinus saxesenii* | Insecta | Coleoptera | Curculionidae | Malaise |
| *Xylotrechus arvicola* | Insecta | Coleoptera | Cerambycidae | Malaise |


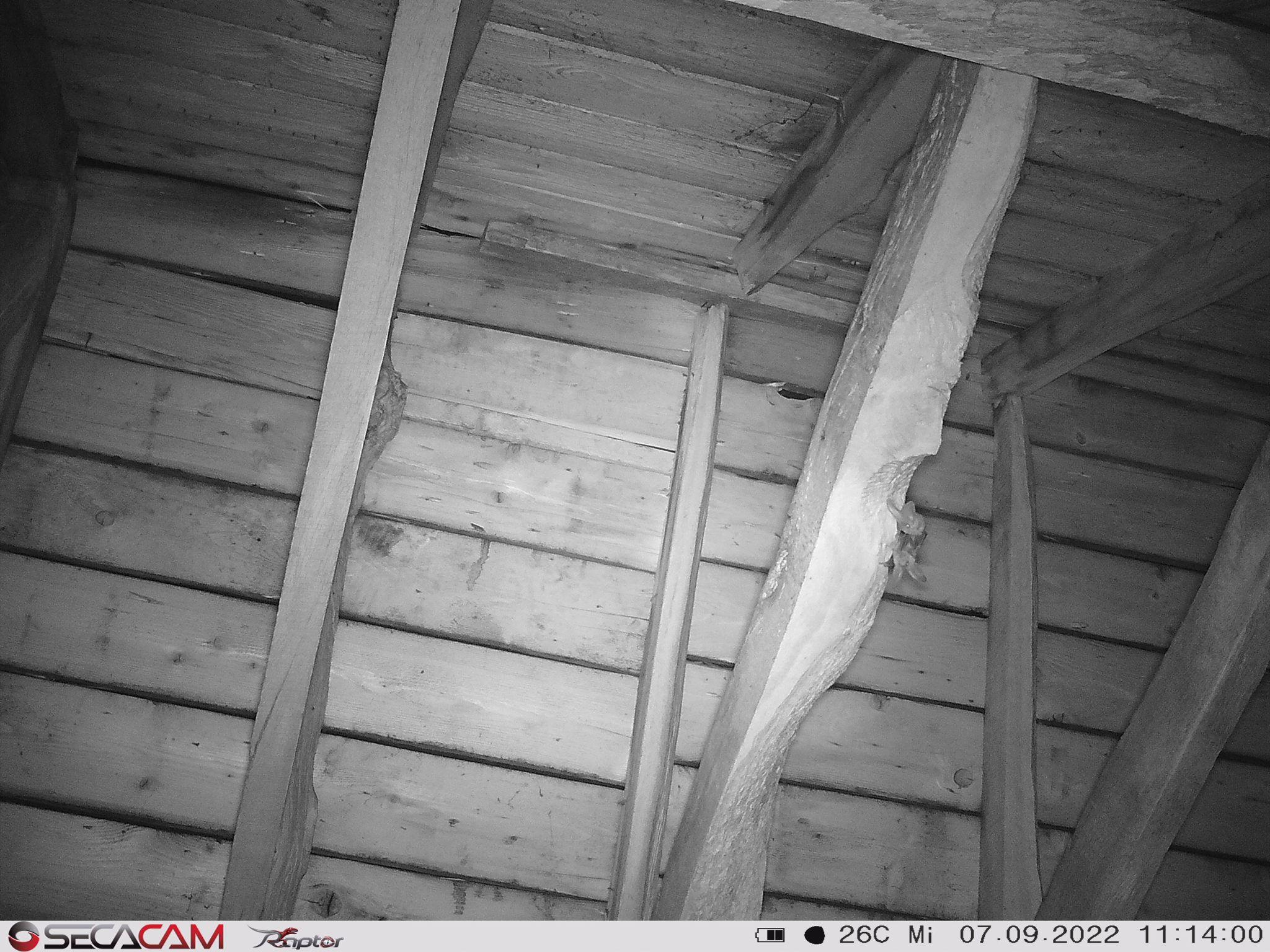

***Figure S1*** - Infrared camera images showing two *Plecotus* sp. individuals roosting on the ceiling of the nave in the Evangelical Church of Flamersheim.


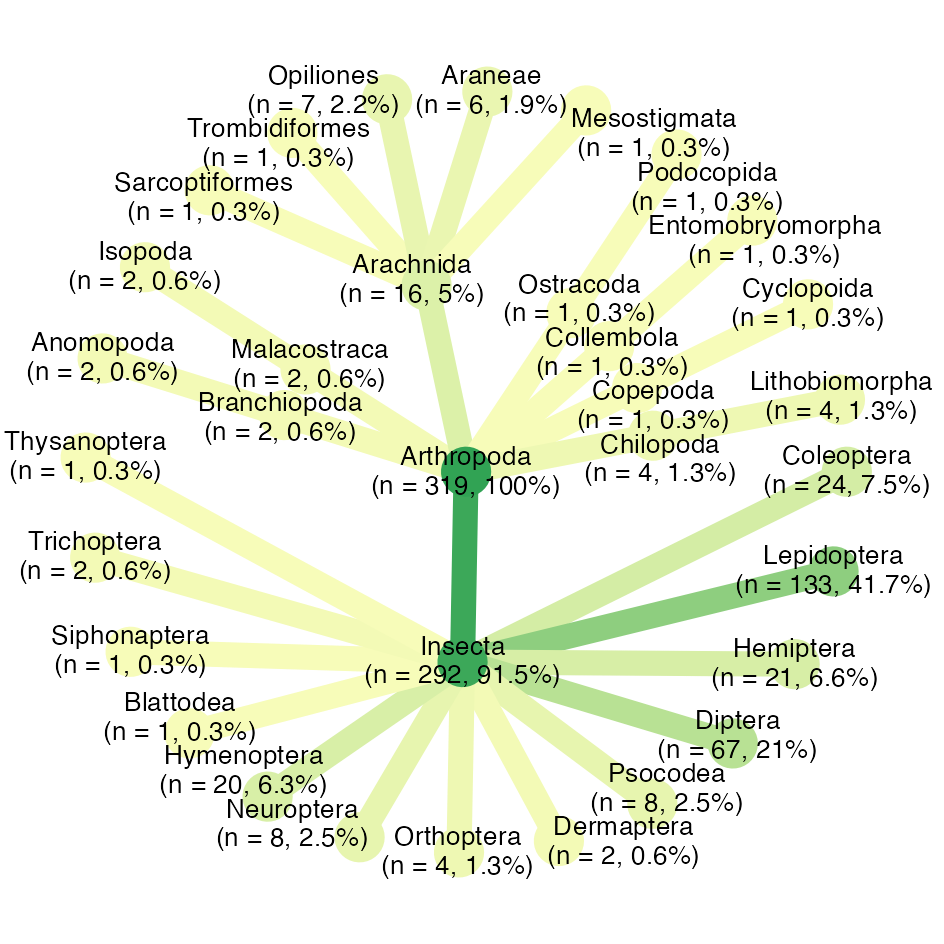


***Figure S2 -*** Number of insect taxa detected in guano samples from both bat species combined.


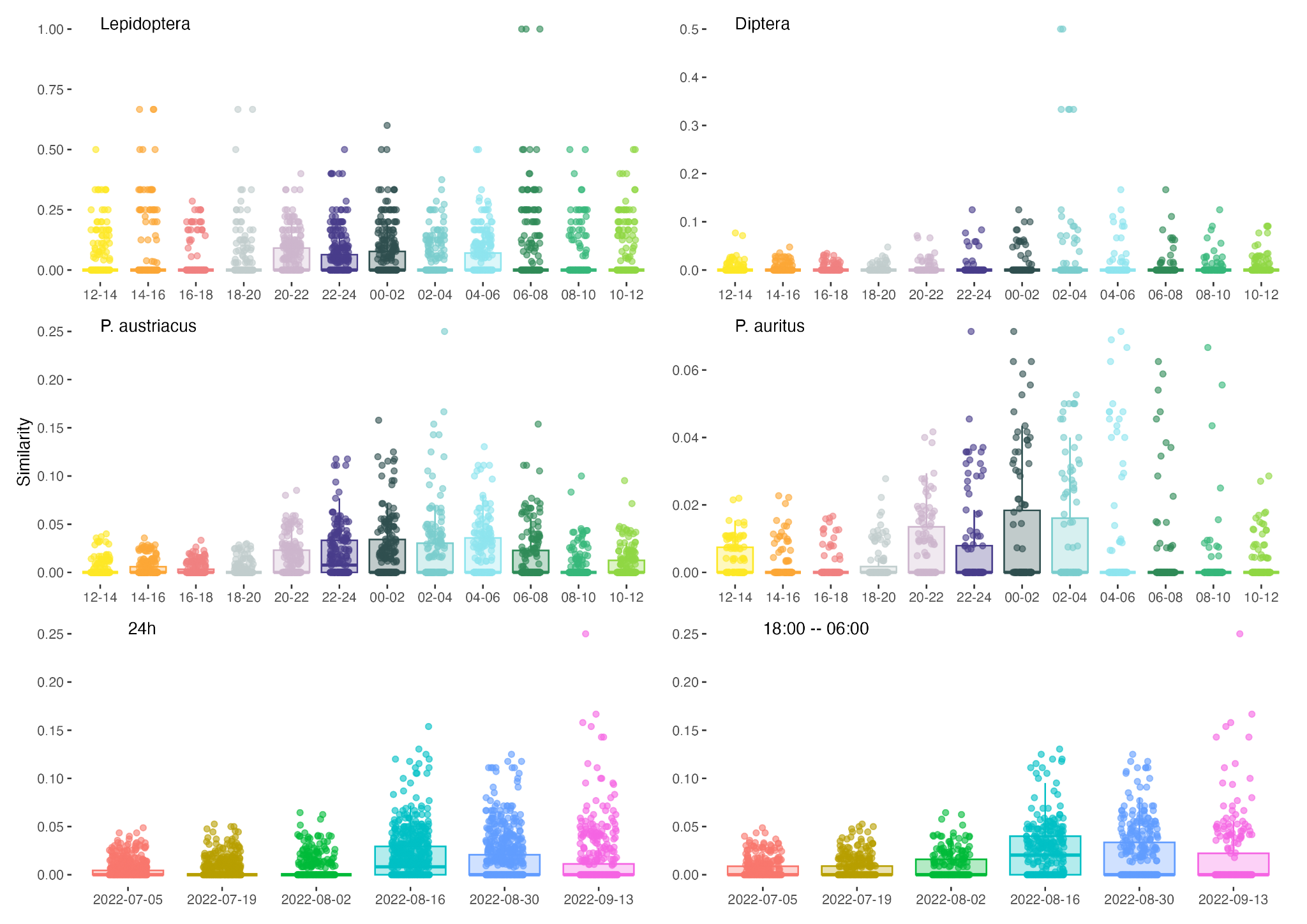

***Figure S3 -*** Arthropod community similarity (1-Jaccard Dissimilarity Index) between guano samples and timed Malaise trap samples. Comparisons were calculated within each sampling round and sampling area using only OTUs identified to species level. Top: subset of Lepidoptera (left) and Diptera (right) species only. Middle: subset of guano samples from P. austriacus (left) and P. auritus (right). Bottom: all comparisons by date considering all time points (left) and only evening and nighttime samples (right). Note different scales on the y-axis.


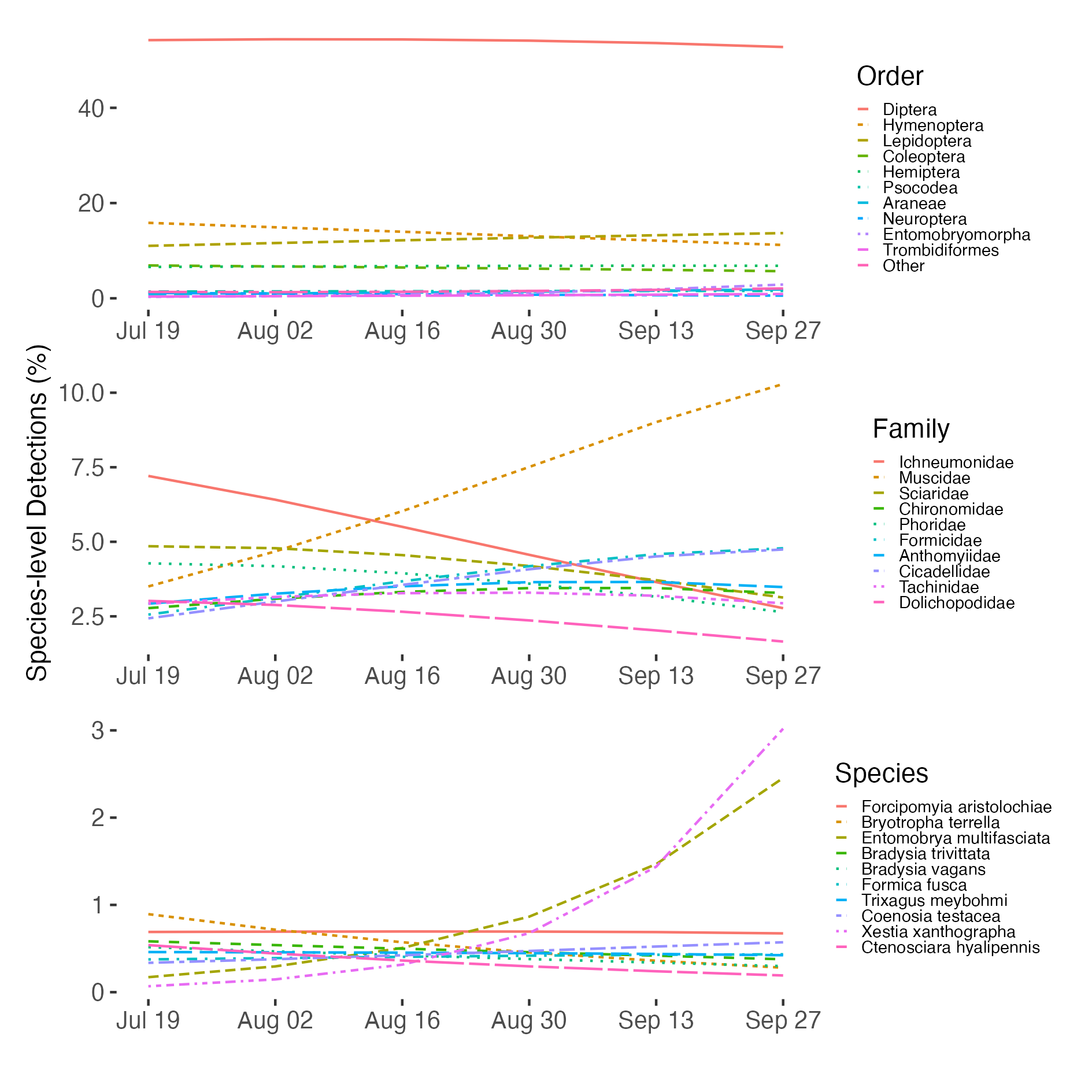


***Figure S4*** - Species-level detections from Malaise traps over time as percent of total detections by order (top) or family (centre), and for the 10 most frequent species (bottom).


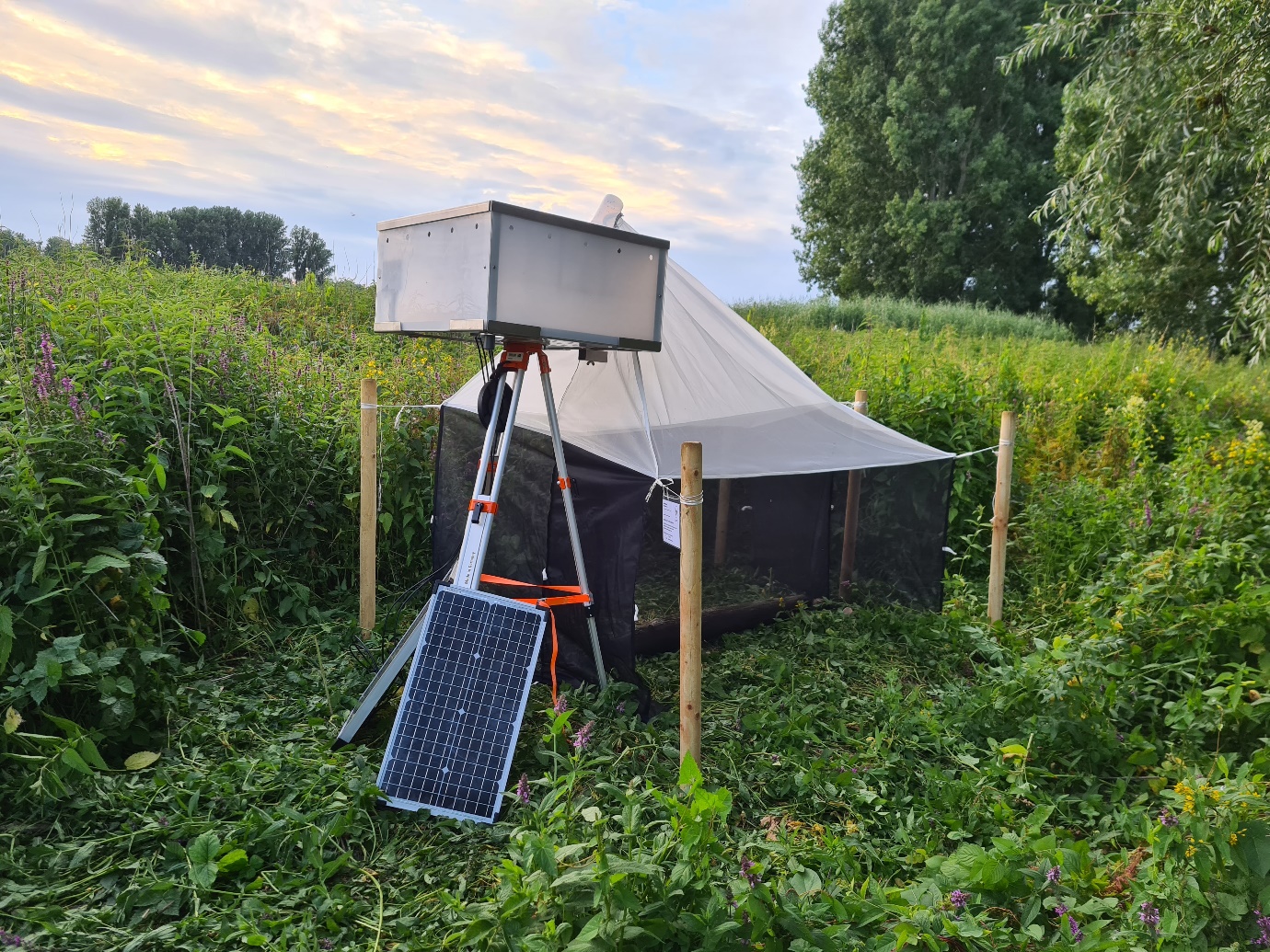

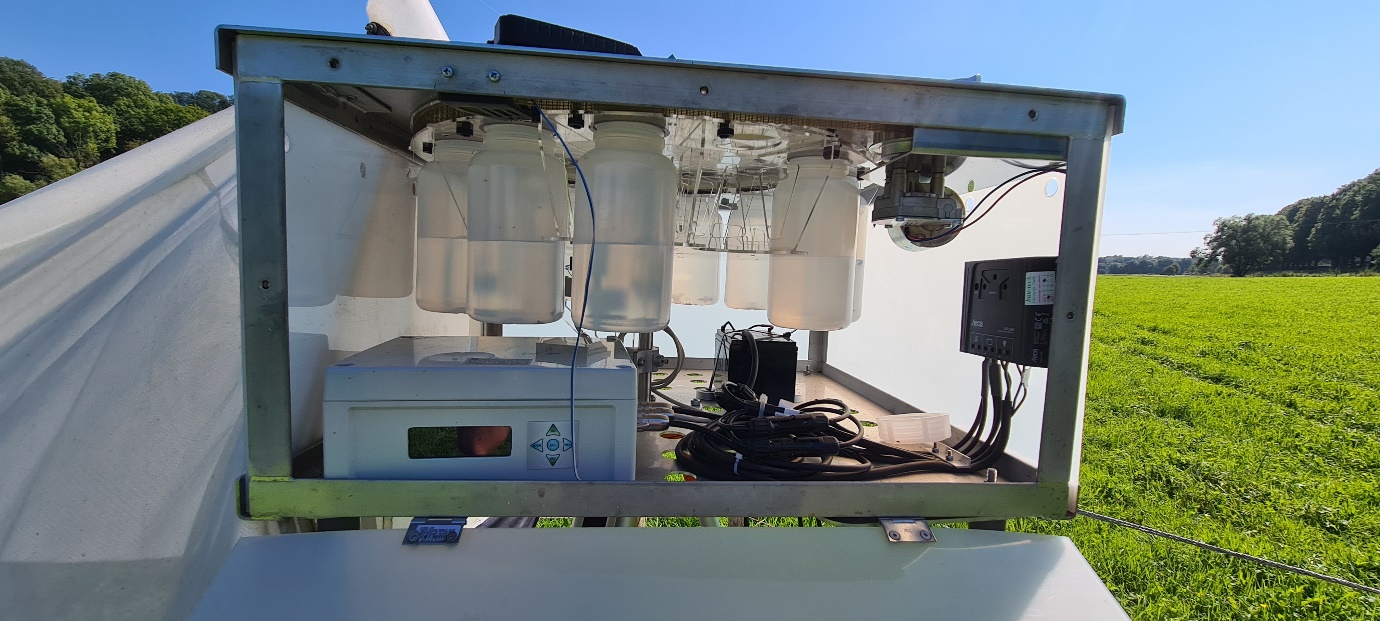


***Figure S5*** – The AMMOD Multisampler installed in the field, and a detailed view of the rotation plate with the screwed‑in collection bottles. Position 13 remains empty to allow for breaks during sampling and to give insects the opportunity to escape during these pauses. For this study, positions 1-12 were equipped with collection bottles. The rotation plate turned every two hours, skipping position 13, enabling continuous monitoring. Each bottle was positioned under the collection head at the exact same time of day across the 14-day sampling period.
